# Genome-Wide Runs of Homozygosity Revealed Selection Signatures in *Bos indicus*

**DOI:** 10.3389/fgene.2020.00092

**Published:** 2020-02-21

**Authors:** S. P. Dixit, Sanjeev Singh, Indrajit Ganguly, Avnish Kumar Bhatia, Anurodh Sharma, N. Anand Kumar, Ajay Kumar Dang, S. Jayakumar

**Affiliations:** ^1^ Animal Genetics Division, ICAR—National Bureau of Animal Genetic Resources, Karnal, India; ^2^ Animal Genetics & Breeding Division, ICAR—National Dairy Research Institute, Karnal, India; ^3^ Animal Physiology Division, ICAR-National Dairy Research Institute, Karnal, India

**Keywords:** autozygosity, genomic inbreeding, runs of homozygosity islands, selection sweep, *F_ROH_*

## Abstract

Genome-wide runs of homozygosity (ROH) are suitable for understanding population history, calculating genomic inbreeding, deciphering genetic architecture of complex traits and diseases as well as identifying genes linked with agro-economic traits. Autozygosity and ROH islands, genomic regions with elevated ROH frequencies, were characterized in 112 animals of seven Indian native cattle breeds (*B. indicus*) using BovineHD BeadChip. In total, 4138 ROH were detected. The average number of ROH per animal was maximum in draft breed, Kangayam (63.62 ± 22.71) and minimum in dairy breed, Sahiwal (24.62 ± 11.03). The mean ROH length was maximum in Vechur (6.97 Mb) and minimum in Hariana (4.04 Mb). Kangayam revealed the highest ROH based inbreeding (*F_ROH_*_>__1Mb_ = 0.113 ± 0.059), whereas Hariana (*F_ROH_*_>__1Mb_ = 0.042 ± 0.031) and Sahiwal (*F_ROH_*_>__1Mb_ = 0.043 ± 0.048) showed the lowest. The high standard deviation observed in each breed highlights a considerable variability in autozygosity. Out of the total autozygous segments observed in each breed except Vechur, > 80% were of short length (< 8 Mb) and contributed almost 50% of the genome proportion under ROH. However, in Vechur cattle, long ROH contributed 75% of the genome proportion under ROH. ROH patterns revealed Hariana and Sahiwal breeds as less consanguineous, while recent inbreeding was apparent in Vechur. Maximum autozygosity observed in Kangayam is attributable to both recent and ancient inbreeding. The ROH islands were harbouring higher proportion of QTLs for production traits (20.68% vs. 14.64%; P≤ 0.05) but lower for reproductive traits (11.49% vs. 15.76%; P≤ 0.05) in dairy breeds compared to draft breed. In draft cattle, genes associated with resistant to diseases/higher immunity (*LYZL1, SVIL,* and *GPX4*) and stress tolerant (*CCT4*) were identified in ROH islands; while in dairy breeds, for milk production (*PTGFR, CSN1S1, CSN2, CSN1S2,* and *CSN3*). Significant difference in ROH islands among large and short statured breeds was observed at chromosome 3 and 5 involving genes like *PTGFR* and *HMGA2* responsible for milk production and stature, respectively. PCA analysis on consensus ROH regions revealed distinct clustering of dairy, draft and short stature cattle breeds.

## Introduction

Runs of homozygosity (ROH), the indicator of genomic autozygosity, may be defined as two contiguous identical by descent (IBD) stretches of homozygous genotypes/segments/haplotypes of a common ancestor in an individual inherited from both of its parents (Gibson et al., 2006).

This autozygosity may arise in inbred as well as non-inbred populations due to several population phenomena like inbreeding, genetic drift, consanguineous mating, population bottleneck, as well as natural and artificial selection (Falconer and Mackay, 1996; Curik et al., 2014). Scanning of genome for ROH using high density SNP arrays in cattle has been found to be effective in discriminating non-autozygotic identical by state (IBS) segments from autozygotic (IBD) (Howrigan et al., 2011). Therefore, the identification and characterization of ROH may help in revealing population structure and demographic history evolved over time as well as unveiling footprints of natural and/or human made selection. The length and frequency of ROH are two important parameters for determining causative forces of genomic change.

Since recombination events interrupt lengthy genomic segments, it is anticipated that longer ROH will appear as a result of recent inbreeding in the pedigree. A negative correlation existed between length of the runs and number of generations back the selection or inbreeding event occurred (Howrigan et al., 2011). Consequently, ROH may be useful for ascertaining signatures of recent and/or ancient selection events (Purfield et al., 2012). Although, recombination events are random and distribution of ROH across samples is likely to be exceptionally heterogeneous; however, selection leaves certain peaks across the genome. These peaks in terms of frequency of ROH are called hotspots and considered to be the signal of selective sweeps (Curik et al., 2014). These hotspots (stretches of homozygous sequences) shared by large proportion of individuals in a population are characterized as ROH islands, the footprints of selection event. ROH may also be an accurate estimator of inbreeding coefficient and has recently been used in calculating inbreeding coefficient of Gyr cattle (Peripolli et al., 2018). The selection sweeps were also studied using ROH information in cattle (Iacolina et al., 2016).

Cattle breeds in India have evolved over the centuries under diverse agro-climatic conditions as well as breeding and management practices for the purpose of different specialized functions such as dairy, draft and dual (dairy and draft). Consequently, these cattle harbouring putative signatures for specific functions may serve a great reservoir of genetic pool for identification of genes under selection particularly those in ROH regions. Here, three dairy breeds (Sahiwal, Gir, Tharparkar) of sub-tropical hot arid regions, two dual breeds viz. Hariana of sub-tropical hot arid region, and Ongole of tropical semi-arid region were considered. One draft breed, Kangayam of tropical semi-arid region, and one short statured cattle breed, Vechur of tropical hot humid region were also included. All these breeds except Vechur are international breeds, and are bred in good number in different countries across the continents.

The present study aimed at delineating autozygosity by identifying and characterizing genome wide ROH patterns in seven Indian native cattle breeds using high density SNP genotyping array (Illumina BovineHD BeadChip). Further, the gene content in ROH regions of these diverse breeds (dairy, dual, draft, large, and small) was also explored to apprehend selection/adaptive footprints.

## Materials and Methods

### Animal Resources, SNP Genotyping, and Quality Control

A total of 132 samples of Sahiwal (SW, n = 19), Tharparkar (TR, n = 17), Gir (GR, n = 16), Ongole (OG, n = 24), Hariana (HR, n = 18), Kangayam (KG, n = 18) and Vechur (VC, n = 20) breeds of cattle were incorporated. The random blood samples were collected from different farms in the country in compliance with the guidelines and regulations of the Institutional Animal Ethics Committee (IAEC), National Bureau of Animal Genetics Resources (ICAR-NBAGR), Karnal (**Supplementary Figure S1**). After isolation of genomic DNA, estimation of quality and quantity was carried out as described earlier (Dash et al., 2018). DNA samples were genotyped at Sandor Lifesciences Pvt. Ltd., Hyderabad, India by using BovineHD BeadChip (Illumina, Inc. San Diego, CA, USA) following standard procedures of the manufacturer. The PLINK v 1.9 (Purcell et al., 2007; Chang et al., 2015) software was used for quality filtration of genotyped data. Only the SNPs located on autosomes were considered for analysis. SNPs that had call rate (CR) ≤ 90%, minor allele frequency (MAF) ≤ 0.05 and HWE (P ≤0.001) were excluded. Further, samples with more than 10% missing genotypes were also omitted.

### Measure of Runs of Homozygosity and Their Distribution

ROH was estimated for each individual using PLINK v 1.9 (Purcell et al., 2007; Chang et al., 2015). Although, no linkage disequilibrium (LD) based pruning was performed; however, the minimum ROH length was set to 1 Mb for excluding short and common ROH that appeared across genome due to LD (Purfield et al., 2012). The following PLINK parameters and thresholds (Purcell et al., 2007) were applied to define a ROH: i) sliding window of 50 SNPs across the genome; ii) proportion of homozygous overlapping windows was 0.05; iii) minimum number of consecutive SNPs included in a ROH was 100; iv) minimum length of a ROH was set to 1 Mb; v) maximum gap between consecutive homozygous SNPs was 1000 kb; vi) a density of one SNP per 50 kb; and vii) maximum of five SNPs with missing genotypes and up to one heterozygous genotype were allowed in a ROH. All ROH were grouped into five classes as per the nomenclature of Kirin et al. (2010) and Ferenčaković et al. (2013a; 2013b): 1–2, 2–4, 4–8, 8–16, and >16 Mb. For every individual in each of the seven breeds, and for each ROH length category, the mean number of ROH per individual (MN_ROH_), the average length of ROH (AL_ROH_) and the total number of ROH per breed (*n*ROH) were estimated. The percentage of chromosomes covered by ROH was also calculated. First, the mean ROH length was calculated by summing all ROH (Mb) on a chromosome and dividing by the number of individuals that had ROH on that chromosome; the mean ROH length was then divided by the length of the chromosome in Mb.

Alternative to PLINK, an R package called “detectRUNS” was additionally used to explore genome wide ROH and the results were compared. It makes use of two methods: 1) sliding-window and 2) consecutive runs. The sliding-window based technique is comparable to PLINK (Purcell et al., 2007); whereas, the consecutive runs is a window free technique to scan the genome SNP by SNP (Marras et al., 2015).

### Genomic Inbreeding Coefficients

PLINK v 1.9 (Purcell et al., 2007; Chang et al., 2015) was used to estimate the genomic inbreeding coefficients (*F_ROH_* and *F_HOM_*). Inbreeding coefficient based on ROH (*F_ROH_*) for each animal was calculated according to McQuillan et al. (2008):

$$F_{\mathrm{ROH}}=\frac{L_{\mathrm{ROH}}}{L_{\mathrm{AUTO}}}$$

where L_ROH_ represents total length of all ROH in an individual genome while L_AUTO_ refers to the autosomal genome length covered by SNPs included in the array. For each animal *F_ROH_* (*F_ROH_*_ > 1Mb_ and *F_ROH_*_ > 8 Mb_) was calculated according to ROH distribution within the length categories: >1 and >8 Mb. Inbreeding based on the observed versus expected number of homozygous genotypes (*F_HOM_*) was calculated using PLINK v1.90 by computing observed and expected autosomal homozygous genotypes counts for each sample as follows:

$$F_{\mathrm{HOM}}=\frac{\mathrm{Number}\;\mathrm{of observed homozygous loci}-\mathrm{Number of expected homozygous loci}}{\mathrm{Number of nonmissing loci}-\mathrm{Number of expected homozygous loci}}$$

Spearman’s correlation coefficients among inbreeding measures (*F_ROH_* and *F_HOM_*) were also estimated.

### Detection and Analyses of Common Runs of Homozygosity

Overlapping ROH were analyzed by PLINK software. Samples were analyzed overall, breed wise, and utility wise (dairy vs. draft). The number of consensus samples was identified in each group and ROH island frequencies were calculated by dividing the number of consensus samples with total samples in each group. To identify genomic regions most commonly associated with ROH, the samples were analyzed using Manhattan plots of overlapping ROH% across the autosomes for each group. Top 20 ROH islands having a frequency of at least 20% were identified in each group from Manhattan Plots. NCBI map viewer of the bovine UMD3.1.1 (https://www.ncbi.nlm.nih.gov/genome/gdv/) was used to identify genes underlying ± 2 MB region on either side of consensus region of top 20 ROH islands. Cattle QTL database (https://www.animalgenome.org/QTLdb/cattle) was explored to find the effect of top 20 ROH islands on the underlying QTLs. Test of two proportions was carried out to find the test of significance between the numbers of QTLs affecting the two contrasting groups (dairy vs. draft) under six different traits using XLSTAT. Top five ROH hot spots from the overall ROH group were explored to find the frequency of ROH islands at analogous positions in each cattle breed. Test of K proportion (XLSTAT) was carried out to find out the significant difference of ROH frequencies among the breeds. Gene ontology and pathway analyses were carried out by PANTHER version 13.1 software tool (http://pantherdb.org). Pathway analysis was also carried out by Reactome pathway (https://reactome.org).

### Structuring of Cattle

Genomic relationship matrix-based principal component analysis (PCA) was performed using the R software “*factoextra*” (https://cran.r-project.org) to better evaluate the composition of the breeds and to define genetic groups for further downstream calculations. Top 170 ROH regions (loci) of the total samples were selected with a frequency of at least 12.5% for PCA. Three components were extracted out of 6 using Kaiser Rule criterion (Johnson and Wichern, 1982) to determine the number of significant components. Further, number of loci contributing maximum to the total variance were scaled down. The different graphs and plots were generated representing the contribution of the loci and individuals to the total genetic variation.

## Results

### Filtration, Polymorphism, and Genetic Diversity Among the Breeds

Out of 132 animals, 20 were removed due to low genotyping (MIND > 0.1). The overall genotyping rate for the remaining 112 animals was 0.99. The quality control measures led to final data on 112 cattle belonging to Sahiwal (13), Tharparkar (17), Gir (15), Ongole (17), Hariana (18), Kangayam (16), and Vechur (16) breeds. Minor allele frequency across the breeds ranged from 0.23 (Kangayam) to 0.26 (Vechur) and observed heterozygosity on an average was 0.35 in all the studied samples (data not shown).

### ROH Distribution and Genomic Inbreeding

**Figure 1:**
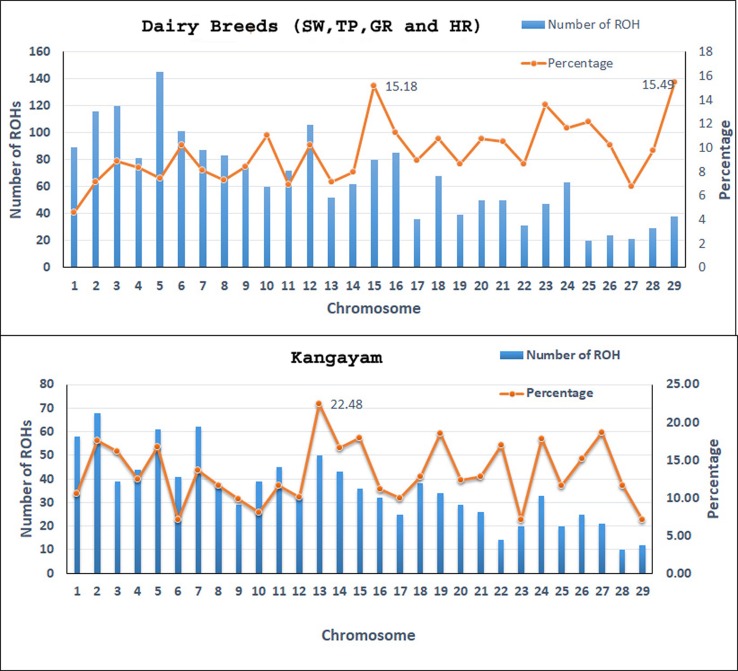
The number of ROH per chromosome and percentage coverage. The bars exhibit the total number of ROH per chromosome identified in the 112 animals. The line shows the average percentage of ROH for every chromosome. To determine the percentage of ROH per chromosome, the mean ROH length was calculated by adding all ROH (in Mb) on a chromosome and then dividing by the number of animals that had ROH on that chromosome. The mean ROH length was then divided by the chromosome length (in Mb) and transformed to percentage. SW, Sahiwal; TP, Tharparkar; GR, Gir; HR, Hariana.

A total of 4138 homozygous segments were identified. The mean number of ROH per animal was highest in draft breed, Kangayam (63.62 ± 22.71 with a range of 11- 92) and lowest in Sahiwal (24.62 ± 11.03 with a range of 12–49). Although, average length of ROH (AL_ROH_) was maximum in Vechur (6.97 Mb) and minimum in Hariana (4.04 Mb) (**Table 1**); however, mean genome length under ROH was highest in Kangayam (283.74 Mb; 11.30%) and lowest in Hariana (106.61 Mb; 4.24%). The longest ROH segment (80.22 Mb harbouring 17050 SNPs) was observed on chromosome 6 in Tharparkar (**Supplementary Table S1**). The highest number of ROH (n= 145) was observed on BTA 5 in dairy (SW, TP, GR) and dual (HR) breeds but on BTA 2 in draft breed (n= 68). Major fraction of chromosome residing in ROH was observed on BTA 29 & BTA 15 (15.49% & 15.18%, respectively) in dairy and dual breeds but on BTA 13 (22.48%) in draft cattle (**Figure 1**). The number and percentage coverage of chromosomal length by ROH varied from breed to breed (**Supplementary Figure S2**).

**Table 1:** Genomic distributions and descriptive statistics of ROH in different *Bos indicus* breeds.

Breeds	*n*ROH	Range ROH	NM_ROH_	MGL_ROH_	MGP_ROH_	AL_ROH_	*F_ROH_*_ > 1 Mb_	*F_ROH_*_ > 8 Mb_	F_HOM_	r(*F_ROH_*_ > 1 Mb_ - *F_HOM_*)	r(*F_ROH_*_ > 8 Mb_ - *F_HOM_*)
Sahiwal	320	12-49	24.62 ± 11.03	107.45	4.28	4.37	0.043 ± 0.048	0.023 ± 0.040	-0.034 ± 0.061	0.939	0.948
Tharparkar	453	10-53	26.65 ± 12.60	134.56	5.36	5.05	0.054 ± 0.048	0.032 ± 0.036	-0.040 ± 0.053	0.954	0.940
Gir	680	15-96	45.33 ± 21.51	211.95	8.44	4.68	0.085 ± 0.093	0.047 ± 0.075	-0.024 ± 0.116	0.950	0.944
Hariana	475	8-42	26.39 ± 7.25	106.61	4.24	4.04	0.042 ± 0.031	0.022 ± 0.028	-0.030 ± 0.036	0.959	0.949
Kangayam	1024	11-92	63.62 ± 22.71	283.74	11.3	4.43	0.113 ± 0.059	0.052 ± 0.038	-0.074 ± 0.111	0.888	0.839
Ongole	761	33-61	44.94 ± 9.54	188.22	7.49	4.20	0.075 ± 0.064	0.037 ± 0.059	-0.027 ± 0.070	0.987	0.979
Vechur	425	07-64	26.56 ± 16.15	185.13	7.37	6.97	0.074 ± 0.080	0.055 ± 0.073	0.034 ± 0.104	0.810	0.843
Dairy Breeds	1928	08-96	30.60± 15.90	139.41	5.55	4.56	0.061 ± 0.063	0.032 ± 0.053	-0.029 ± 0.074	0.925	0.936

*n*ROH: Total number of ROH per breed; NM_ROH_: Mean number of ROH in a breed; MGL_ROH_: Breed wise mean genome length covered by ROH in Mb; MGP_ROH_: Breed wise mean genome proportion covered by ROH in percentage; AL_ROH_: Average length of ROH (Mb) in a breed. Dairy Breeds: Sahiwal, Tharparkar, Gir, Hariana. *F_ROH_*: Inbreeding coefficient based on ROH; *F_HOM_*: Inbreeding coefficient based on homozygous loci; r: spearman’s correlation coefficient.

**Figure 2:**
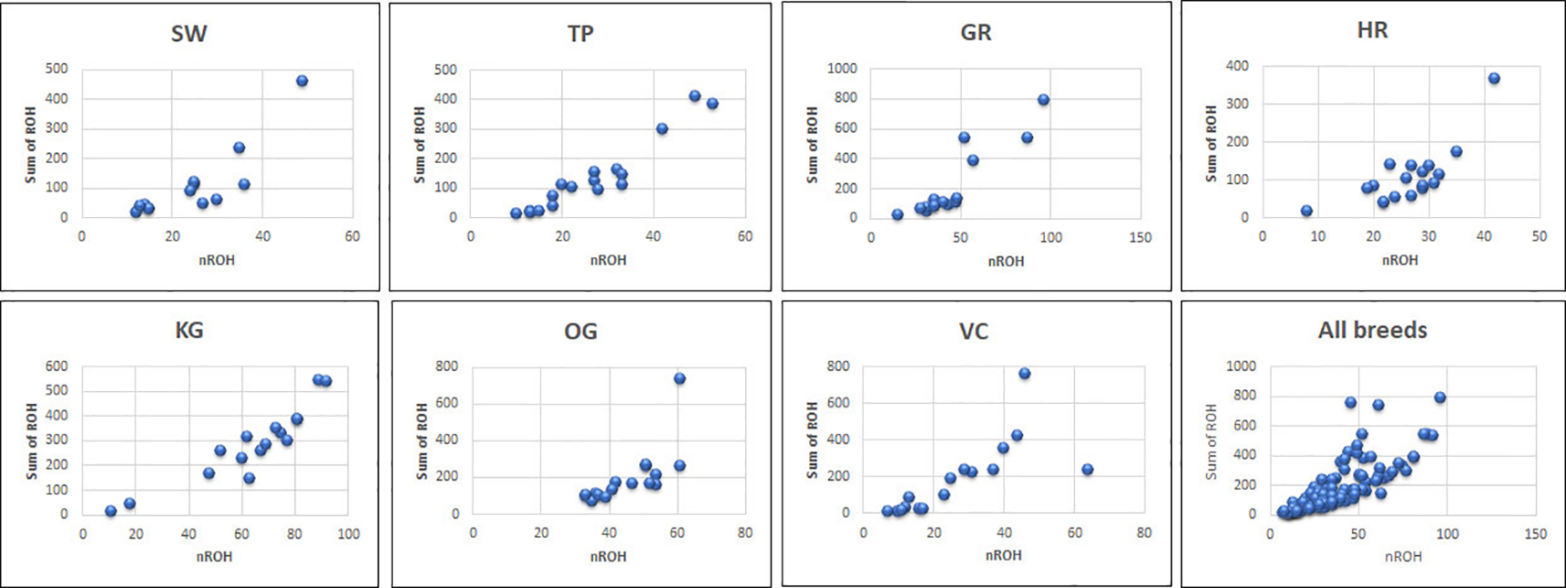
The total number of ROH and length of genome under ROH for each individual in a breed. SW, Sahiwal; TP, Tharparkar; GR, Gir; HR, Hariana; KG, Kangayam; OG, Ongole; VC, Vechur.

**Figure 3:**
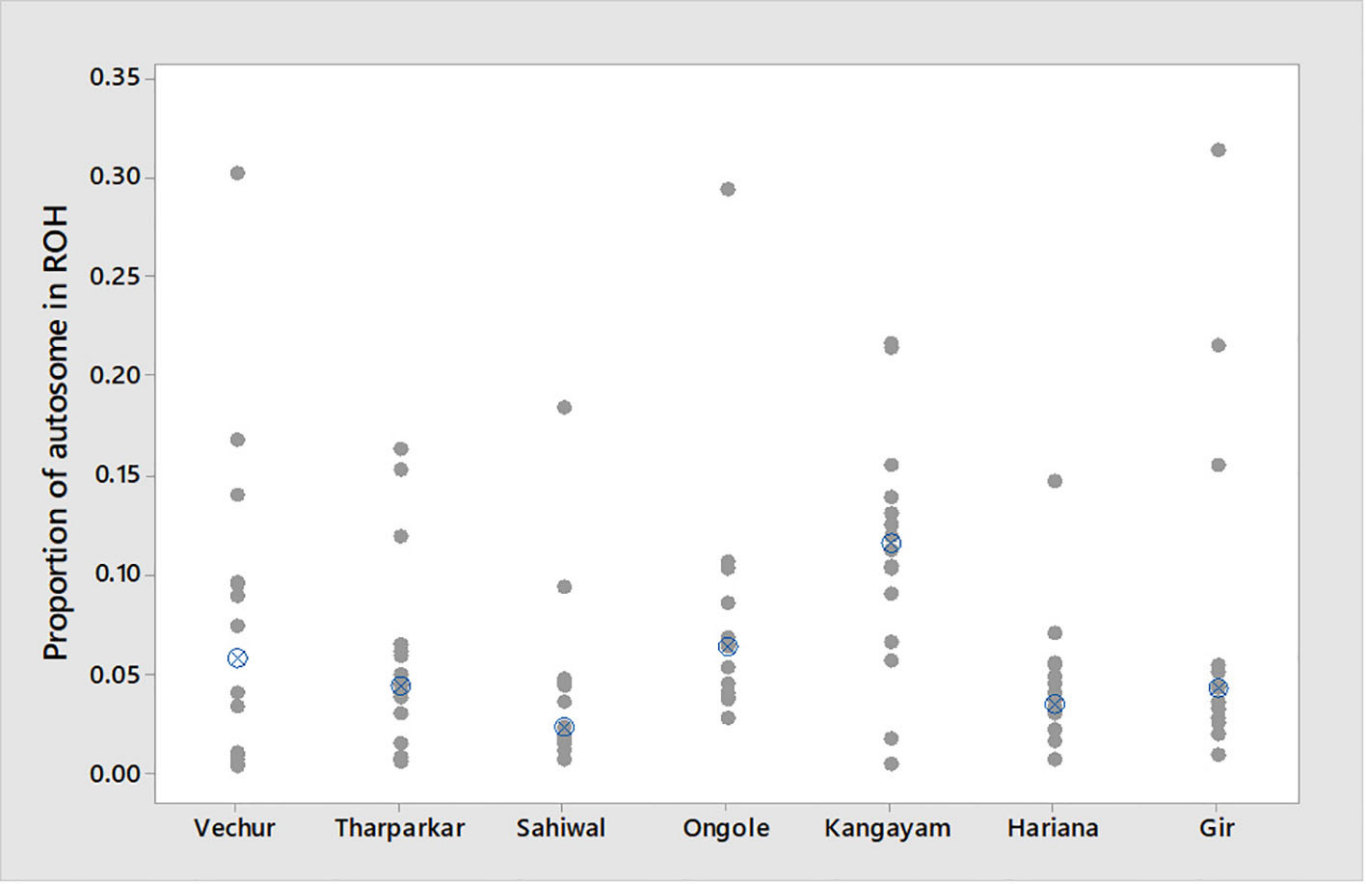
Individual value plot displaying proportion of autosome covered in runs of homozygosity (ROH) per animal. The crossed circle shows the median ROH value of each breed.

**Figure 4:**
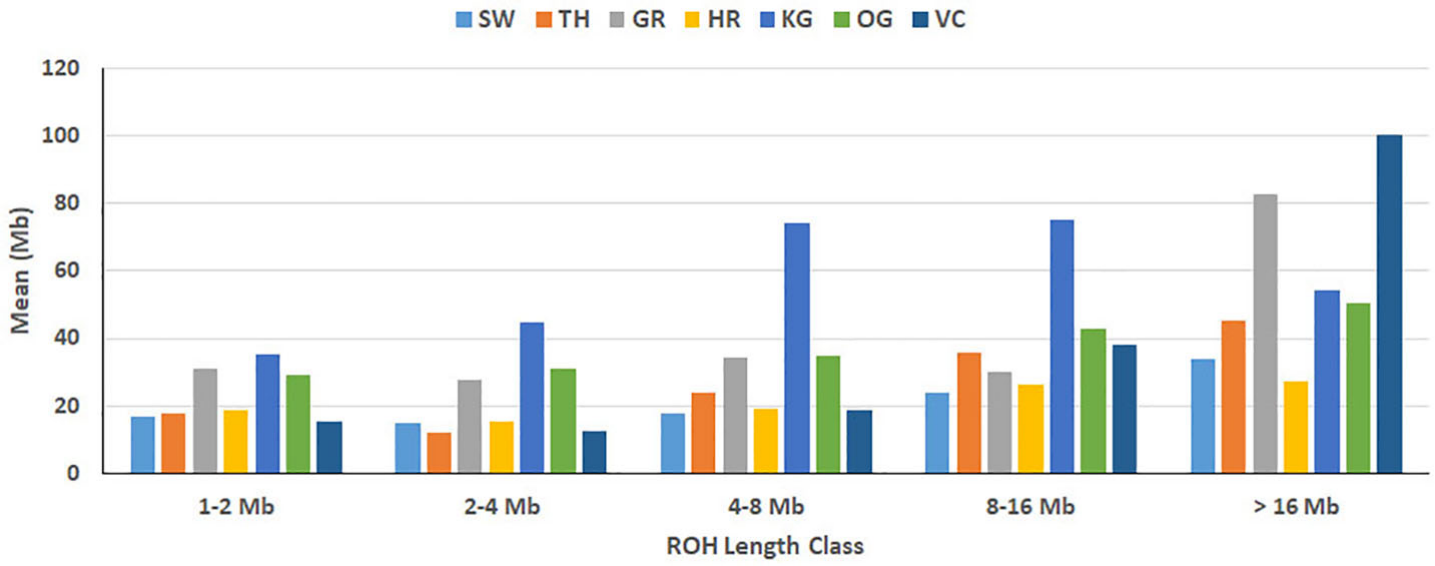
Breed-wise mean of sum of ROH. Within each ROH length category, the sum of ROH (in Mb) was calculated per animal and averaged breed-wise. Breeds from left to right are Sahiwal (SW), Tharparkar (TP), Gir (GR), Hariana (HR), Kangayam (KG), Ongole (OG), and Vechur (VC).

The total number and length of genome under ROH for each individual in a breed are presented in **Figure 2**. The majority of the individuals (69.64%) clustered close to the origin of coordinates due to abundance of shorter ROH (**Figure 2**). The total length of ROH across genome was <176 Mb in most of the individuals (84.38%) in dairy breeds but varied between 200-400 Mb in draft breed (62.5%) (**Figure 2**). There were seven individuals with ROH length between 400 to 550 Mb and three individuals (one each from GR, OG, and VC) with more than 700 Mb of their autosomes covered by ROH (**Supplementary Table S1**). The proportion of the autosome under ROH varied both within and between breeds (**Figure 3**). Sahiwal had a tendency towards smaller proportion of genome under ROH but Kangayam towards larger. The later showed higher inter-animal variability (12.63 Mb to 543.81 Mb). All the 112 individuals of seven cattle breeds had at least one ROH in 1–2 Mb category. About 95% animals had at least one ROH between 2 and 4 Mb in length. The frequency of ROH in the different categories varied among the breeds (**Figure 4**, **Table 2**). Out of the total autozygous segments observed in each breed except Vechur, > 80% of the ROH were of short length (< 8 Mb) and contributed almost 50% of the genome coverage of ROH under this category. However, in Vechur cattle, long ROH (> 8 Mb) contributed 75% of the genome coverage under ROH (**Tables 1** and **2**).

**Table 2:** Statistics of ROH observed in diverse Indian native cattle breeds (*Bos indicus*) under different length class (ROH_1-2 Mb_, ROH_2-4 Mb_, ROH_4-8 Mb_, ROH_8-16 Mb,_ ROH_ > 16 Mb_, ROH_ > 1 Mb_ and ROH_ > 8 Mb_).

Breeds	Sahiwal	Tharparkar	Gir	Hariana	Kangayam	Ongole	Vechur
*n* **ROH**	320	453	680	475	1024	761	425
**ROH_1-2 Mb_** ** *N*ROH (percent)** **Mean length (Mb) ± SD** **Genome coverage (%)**	172 (53.75) 1.29 ± 0.26 0.68	231 (50.99) 1.30 ± 0.27 0.70	342 (50.30) 1.42 ± 0.27 1.24	254 (53.47) 1.32 ± 0.25 0.74	411 (40.14) 1.36 ± 0.30 1.40	365 (47.96) 1.35 ± 0.27 1.15	188 (44.24) 1.30 ± 0.25 0.61
**ROH_2-4 Mb_** ** *N*ROH (percent)** ** Mean length (Mb) ± SD** ** Genome coverage (%)**	65 (20.31) 2.97 ± 0.61 0.59	72 (15.89) 2.83 ± 0.58 0.48	153 (22.50) 2.79 ± 0.52 1.11	98 (20.63) 2.83 ± 0.57 0.612	251 (24.51) 2.87 ± 0.58 1.79	189 (24.84) 2.80 ± 0.59 1.24	73 (17.18) 2.79 ± 0.57 0.51
**ROH_4-8 Mb_** *** N*ROH (percent)** ** Mean length (Mb) ± SD** ** Genome coverage (%)**	40 (12.50) 5.72 ± 1.15 0.70	67 (14.80) 6.12 ± 1.18 0.96	91 (13.38) 5.86 ± 1.12 1.37	62 (13.05) 5.58 ± 1.21 0.76	212 (20.70) 5.61 ± 1.10 2.96	107 (14.06) 5.45 ± 1.07 1.38	52 (12.24) 5.73 ± 1.13 0.74
**ROH_8-16 Mb_** *** N*ROH (percent)** ** Mean length (Mb) ± SD** ** Genome coverage (%)**	26 (8.13) 11.93 ± 1.87 0.95	55 (12.14) 11.01 ± 2.41 1.42	48 (7.06) 12.86 ± 2.23 2.39	42 (8.85) 11.30 ± 2.36 1.05	113(11.04) 10.65 ± 2.06 2.99	66 (8.67) 11.09 ± 2.20 1.71	56 (13.17) 10.92 ± 1.97 1.52
**ROH_ > 1__6 Mb_** *** N*ROH (percent)** ** Mean length (Mb) ± SD** ** Genome coverage (%)**	17 (5.31) 26.07 ± 9.38 1.36	28 (6.18) 27.44 ± 13.51 1.80	46 (6.76) 26.32 ± 8.8 3.28	19 (4.00) 25.60 ± 9.53 1.08	37 (3.61) 23.41 ± 8.34 2.16	34 (4.47) 25.26 ± 9.70 2.01	56 (13.17) 28.14 ± 10.02 3.99
**ROH_ > 1__ Mb_** *** N*ROH (percent)** ** Mean length (Mb) ± SD** ** Genome coverage (%)**	277 (86.56) 2.32 ± 1.66 1.97	370 (81.68) 2.47 ± 1.92 2.14	586 (86.18) 2.39 ± 1.62 3.72	414 (87.16) 2.31 ± 1.61 2.12	874 (85.35) 2.41 ± 1.82 6.14	661 (86.86) 2.43 ± 1.59 3.77	313 (73.65) 2.38 ± 1.71 1.86
**ROH_ > 8__ Mb_** *** N*ROH (percent)** ** Mean length (Mb) ± SD** ** Genome coverage (%)**	43 (13.44) 17.52 ± 9.19 2.30	83 (18.32) 16.55 ± 11.18 3.21	94 (13.82) 18.90 ± 10.13 4.72	61 (12.84) 15.76 ± 8.7 2.13	150 (14.65) 13.80 ± 7.1 5.15	100 (13.14) 15.91 ± 8.95 3.72	112 (26.35) 19.78 ± 11.44 5.51

Average genomic inbreeding (*F_ROH_*_> 1 Mb_) coefficient of Kangayam cattle was higher compared to that of Gir, Ongole and Vechur. On the other hand, *F_ROH_*_> 8Mb_ of Vechur was higher than that of Kangayam and Gir. However, the inbreeding coefficient of Hariana and Sahiwal was lower as compared to other breeds. The correlations of *F_HOM_* with *F_ROH_*_> 1 Mb_ and *F_ROH_*_> 8 Mb_ ranged from 0.810 to 0.959 (*F_HOM_* with *F_ROH_*_> 1 Mb_) and 0.839 to 0.979 (*F_HOM_* with *F_ROH_*_> 8 Mb_) across the breeds. (**Table 1**). The genomic inbreeding (*F_ROH_*_> 1 Mb_) values of different breeds/groups are presented in **Supplementary Figure S3**. The slidingRUNS results were similar to the PLINK output (**Table 2**). On the contrary, slight variation in consecutiveRUNS results were observed because of different algorithm (SNP by SNP approach) being used. However, *F_ROH_* calculated using PLINK, consecutiveRUNS and slidingRUNS revealed similar patterns in all the breeds (**Supplementary Figure S3**).

### Genomic Regions Within Overlapping ROH

Principal component analysis based on entire SNP data clustered Hariana, traditionally defined as a dual breed, with other dairy breeds (**Supplementary Figure S4**). Hence in group wise analysis, Hariana was included in dairy group. PCA based clustering was in consonance with breeding of Hariana for higher milk production at the farm for several generations.

The Manhattan plots of overlapping ROH % for each group are presented in **Supplementary Figure S5**. The top 20 ROH islands of dairy breeds (SW, TH, GR, and HR) were harboring significantly (P≤ 0.05) higher proportion of QTL influencing production traits but lower proportion for reproduction traits compared to the draft breed (KG) (**Table 3**). The proportion of QTL in ROH islands were also higher for milk production but lower for meat and carcass traits, however, the differences were non-significant between both the groups. The frequency of top five ROH islands across different chromosomes in each breed indicated significant breed differences at chromosome 3, 5, and 12 (**Table 4**). The ROH islands in Vechur cattle were absent at chromosomes 3 and 5. The genes identified in these regions were *PTGFR* (Prostaglandin F Receptor) and *HMGA2* (High Mobility Group AT-Hook 2) responsible for the milk production and short stature, respectively. There were also significant differences between Gir and Hariana cattle for enriched ROH islands. The detailed functional annotation of genes identified in top 20 ROH islands of dairy and draft breeds is presented in supplementary file (**Supplementary Table S2**). Some of the important genes identified in draft cattle (KG) were *SVIL* (Supervillin), *LYZL1* (Lysozyme like1), *ZEB1* (Zinc Finger E-Box Binding Homeobox 1), and *GPX4* (Glutathione Peroxidase 4); and those in dairy breeds were *PTGFR*, *ZAR1L* (Zygote arrest-1 like), *IFI44* (interferon-induced protein 44), and *HELB* (DNA helicase B).

**Table 3:** Percentage of total QTLs underlying top 20 ROH islands in dairy and draft breeds.

*Trait*	*Dairy breed*	*Draft breed*	*QTL proportion in Dairy breed*	*QTL proportion in* Draft breed	*P value (continuous)*	*P value MCMC with 5000 simulations*
*Health*	39	71	7.47	9.90	0.157	0.133
*Meat and carcass*	101	159	19.34	22.17	0.252	0.223
*Dairy production*	163	200	31.22	27.89	0.229	0.206
*Production*	108	105	20.68	14.64	0.008	0.006*
*Reproduction*	60	113	11.49	15.76	0.035	0.028*
*Exterior*	51	69	9.77	9.62	1.0	0.95
*Total no. of QTLs*	522	717				

Dairy Breeds: Sahiwal, Tharparkar, Gir, Hariana; draft breed-Kangayam; *indicate significant difference (P ≤ 0.05).

**Table 4:** Test of K proportion for the top five ROH hot spots (%) in different breeds of cattle based on overall samples.

*Chromosome Number*	*Physical Position*	*TP*	*SW*	*GR*	*OG*	*HR*	*VC*	*KG*	*Average*	*Genes identified*
*23*	169267-1050607	29.41	23.07	20.00	29.41	22.23	18.75	62.50	29.34	–
***12***	28550326-28754474	17.65	23.07	**60.00**	41.18	**5.55**	12.50	37.50	28.21	NHBP2L2/L1, BCA2, ZAR1L
*7*	45249073-45625200	17.65	30.77	46.67	11.76	11.11	12.50	43.70	24.89	–
***5***	48008400-48069099	**47.05**	30.77	20.00	35.29	11.11	**0**	37.50	25.96	HMGA2,mir763
***3***	66422810-66653007	41.12	23.07	13.33	**47.05**	33.33	**0**	12.50	24.34	PTGFR

Bold face indicates significant difference among breeds (P ≤ 0.05). Sahiwal (SW), Tharparkar (TP), Gir (GR), Hariana (HR), Kangayam (KG), Ongole (OG), and Vechur (VC).

Gene ontology (GO) analysis identified several enriched GO terms for the ROH gene list in dairy as well as draft cattle (**Table 5**).The detailed functions of genes identified from top 20 ROH islands ( ± 2MB) of two groups (dairy and draft) are given in **Supplementary Table S3**. Whereas, genes in the enriched GO and pathways analyses are shown in **Supplementary Table S4**. In both dairy and draft cattle, the G- coupling receptor signaling pathway (**Table 5**) harboring genes for stimuli, smell, cellular defense response and immune system process were under represented. Panther molecular and reactome pathways were not significantly enriched for any specific category of dairy cattle. However, in draft cattle (Kangayam) two reactome pathways were significantly affected viz., activation of pre-replicative complex with a fold difference of 6.91 (P< 4.02 x10^-2^) and G2/M transition with a fold difference of 3.14 (P< 4.35 x10^-2^) (**Table 5**). A total of seven and 16 genes were observed in the two groups, respectively.

**Table 5:** Gene ontology and reactome pathway analyses for the enrichment of GO and reactome pathway terms in dairy and draft cattle.

Term enriched	Number of genes in reference database (*B. taurus*)	Observed number of genes	Expected number of genes	Fold enrichment	+/-	False declaration rate (FDR)
**Dairy cattle**						
**GO term enriched**						
*Panther GO-Slim Biological process*						
Mammary gland development	5	3	0.10	31.41	+	2.08E-02
Steroid metabolic process	115	13	2.20	5.92	+	2.15E-04
G-coupling receptor signaling pathway	753	2	14.38	0.14	-	8.46E-03
*Panther GO Slim cellular component*						
Cell junction	129	11	2.46	4.46	+	2.19E-03
*GO cellular component complete*						
Golgi lumen	10	5	0.19	26.18	+	9.23E-04
**Draft cattle**						
**GO term enriched**						
*Panther GO SLIM biological process*						
G-coupling receptor signaling pathway	753	7	24.59	0.28	-	1.28E-02
*Panther GO SLIM Cellular component*						
Microtubule	145	13	4.74	2.75	+	2.52E-02
*Panther GO Molecular function complete*						
Catalytic activity acting on RNA	363	30	11.85	2.53	+	4.91E-02
Catalytic activity	6041	252	197.27	1.28	+	2.50E-02
**Reactome pathway term enriched**						
Activation of the pre-replicative complex	31	7	1.01	6.91	+	4.02E-02
G2/M transition	156	16	5.09	3.14	+	4.35E-02

FDR < 0.05; ‘+’: Over representation; ‘-’: Under representation.

#### Dairy Breed

In dairy cattle, several genes related to mammary gland development were observed in highly enriched GO terms for biological process, metabolic process and cellular component (**Table 5**). For the biological process, Kappa-casein (*CSN3*) and *COP9* signalosome complex subunit 3 (*COPS3*) were found with highest fold enrichment (31.41). Similarly, 13 genes of steroid metabolic process had an enrichment of 5.92. Panther GO slim cellular component revealed 11 genes with a fold enrichment of 4.46 for cell junction. The key genes were from Cadherin (*CADH1*, *CADH3*, *CADH5,* and *CADH11*) and Myosin (*MYO1A* and *MYO1B*) families. GO cellular component complete revealed five genes with a fold enrichment of 26.18 for Golgi lumen.

#### Draft Breed

Under cellular component (microtubule), 13 genes were involved in cytoskeleton structuring and microtubular functions. 30 genes with catalytic activity acting on RNA (fold enrichment 2.53) and 252 genes with catalytic activity (1.28 fold) were also observed under GO molecular function complete (**Table 5**). In this group, *LYZL1* (Lysozyme like 1; associated with body defense mechanism and disease resistance) as well as genes like *CYB561* (Cytochrome B561) and *GSR* (glutathione reductase, mitochondrial), involved in oxidoreductase activity and antioxidant property, respectively were observed.

### Structuring of Cattle

The structuring of native cattle breeds based on top 170, 92, and 10 contributing ROH islands is presented in **Supplementary Figure S6**. When the number of loci (consensus ROH regions) contributing maximum to the total variance was scaled down from 170 to 92 and 10, similar results were obtained. First three component explained 99% of cumulative variation in the data with first component of PCA explaining 58% of the total variation (**Supplementary Figure S6c**). The analysis revealed that Kangayam and Tharparkar contributed maximum to the total variance followed by Vechur, Gir, Sahiwal, Hariana, and Ongole. Kangayam, being a draft breed, was most distinct from rest of the breeds. The dwarf breed, Vechur was also separated from rest of the breeds. All other breeds viz. Sahiwal, Gir, Tharparkar, Hariana, and Ongole were having their own identities and could not be clustered together.

## Discussion

### ROH Distribution and Genomic Inbreeding

In the present investigation, BovineHD BeadChip was used to characterize autozygosity and ROH islands in seven Indian native cattle breeds (*B. indicus*). Minimum of 13 (SW) to maximum 18 (HR) animals per breed remained after quality filtration. For diversity analysis, the existing number of samples in each breed greater than 12 was adequate and in consonance with other studies (Upadhyay et al., 2017; Colli et al., 2018). It has also been indicated that sample size as small as 4 - 6 (Willing et al., 2012) and polymorphic SNP filtration (Colli et al., 2018; Utsunomiya et al., 2019) can mitigate ascertainment bias as long as the number of markers is sufficiently large as those under the current investigation. Earlier, in Indian dairy cattle breeds, we also tested bovine high-density genotyping array to assess its feasibility for Zebu cattle genomic studies (Dash et al., 2018). The genome proportion under autozygosity was almost equal in short and long ROH in all the breeds except Vechur. The autozygosity ranged from 4.24%–11.3% of the genome. This highlighted low ancient and recent autozygosity in Indian cattle. The results also indicated relatively more intense selection in draft than in dairy and dual breeds due to higher number and genome coverage of ROH. Similar level of genomic autozygosity (7.01%) was also observed in Brazilian Gyr cattle (Peripolli et al., 2018). In Vechur, 7.37% of the total genomic proportion was under ROH, and longer runs (> 8 Mb) were observed to be 26.35% among all the identified segments covering 5.5% of the genome (**Table 2**). The length of ROH is considered to have negative correlation with the time of co-ancestry because random recombination events interrupt lengthy chromosomal segments over a period of time. Hence, long (> 8 Mb) ROH in Vechur might have arisen as result of current inbreeding up to 5 generations (Howrigan et al., 2011; Mastrangelo et al., 2017) and/or bottleneck in this population in recent past.

The larger proportion of genome under longer ROH segments was in consonance with relatively higher inbreeding coefficient of *F_ROH_*_ > 8__Mb_ as well as recent history of Vechur cattle. The Vechur was extensively crossed with exotic breeds like Jersey, American Brown Swiss and Holstein to produce Sunandini, a crossbred population. Subsequently, very few purebred Vechur cattle, sampled for the present study, were maintained in different farms in Kerala state of the country and hence, the inbreeding.

On the contrary, IBD genomic segments from remote ancestors yield short ROH (~1- 8 Mb) revealing a greater historical relatedness (Howrigan et al., 2011) and/or selection (Peripolli et al., 2018). Present results highlighted a lower recent inbreeding compared to ancient inbreeding (**Table 1**) in all the breeds and hence, these breeds are less consanguineous. Three individuals (one each from GR, OG and VC) had more than 700 Mb of their autosomes covered by ROH. Similar to the present findings, comparable genome-wide distributions of ROH in Spanish goat breeds have been reported (Manunza et al., 2016). In commercial sheep, few individuals have also been observed to carry ROH of >600 Mb of their autosome equivalent to almost one-fourth of their genome (Mastrangelo et al., 2017; Purfield et al., 2017).

The genomic inbreeding was generally low (*F_ROH_*_> 1Mb_) in all the breeds except Kangayam (*F_ROH_*_ > 1Mb_ = 0.113). The estimates of inbreeding were in agreement with the abundance and length of ROH in the sampled populations. Estimation of inbreeding coefficients using ROH >8 Mb confirmed to be the most consistent with pedigree-based estimates (Keller et al., 2011; Purfield et al., 2012; Marras et al., 2015), which capture recent inbreeding, and are more accurate (Curik et al., 2014; Kim et al., 2015). Hence, it may reasonably be inferred that these populations are by and large outbred in nature. *F_HOM_* estimates were also low but negative except in Vechur and again confirmed that they are less inbred than the average population (Wang, 2014). *F_ROH_* reveals homozygosity level independent of allele frequencies; whereas, *F_HOM_*is influenced by allele frequencies and consequently by sampling (Zhang et al., 2015). The present estimates corroborated the previous findings in cattle (Zhang et al., 2015; Mastrangelo et al., 2017) and sheep (Purfield et al., 2017). The ancient rate of inbreeding (*F_ROH_*_> 1Mb_) in some of the present breeds (Sahiwal, Hariana, Tharparkar) was similar but higher for others (Gir, Kangayam, Ongole and Vechur) compared to the estimates of Nellore cows (Zavarez et al., 2015). Nevertheless, in Nellore cattle, the recent inbreeding rate (F*_ROH_*_> 8 Mb_) was lower than the current estimates. The average autosomal F*_ROH_*_> 1Mb_ for *Bos taurus* breeds ranged from 6-15% (Ferenčaković et al., 2013b). The high correlations of *F_HOM_* with *F_ROH_*_> 1 Mb_ and *F_ROH_*_> 8 Mb_ across the breeds revealed that the present estimates of inbreeding in these local cattle are almost free from sampling bias. The genome-wide distribution of ROH, its abundance and length revealed that these cattle had not experienced much inbreeding and/or selection pressure as selection increases the accumulation of ROH in the genome and reduces heterozygosity (Karimi, 2013; Marras et al., 2015; Peripolli et al., 2018). The demographic history of other cattle breeds has also been delineated by using ROH information (Bosse et al., 2012).

### Genomic Regions Within Overlapping ROH

The dairy and draft cattle breeds had contrasting phenotypic/production and reproduction characteristics. Kangayam had lower age at first calving (39.99 months) than dairy breeds (mean of 45.09 months for four dairy breeds) indicating its higher reproductive efficiency. Whereas, milk production was lower in Kangayam (540 kg/lactation) compared to the average milk production of 4 dairy breeds (1792.75 Kg/lactation) indicating the superiority of dairy breeds for milk production (http://www.nbagr.res.in/). The production/reproduction characteristics of these breeds were in consonance with the proportion of identified QTL in top 20 ROH in each group for these traits (**Table 3**). The short stature of Vechur could be due to *HMGA2* polymorphism observed in this cattle. *HMGA2* polymorphism had earlier been found to be associated with the difference in body stature in mice (pygmy size) (Zhou et al., 1995), humans (oversize) (Ligon et al., 2005) and cattle (Pryce et al., 2011). Recently, the intronic copy number variation (CNV) of this gene has also been associated with navel length in Nellore cattle (Aguiar et al., 2018).The analysis of the annotated genes in these ROH regions of dairy and draft breeds also indicated that Kangayam was more resistant to diseases/had higher immunity (selection sweeps in *LYZL1*, *SVIL* and *GPX4*) and stress tolerant (*CCT4*). Whereas, dairy breeds had selection sweeps in key genes governing milk production (*PTGFR*, *CSN1S1, CSN2, CSN1S2,* and *CSN3*).

Besides *CSN3* and *COPS3,* cadherin and myosin family genes were found to be enriched under GO Slim cellular component (cell junction) in dairy breeds, indicating their explicit role in mammary gland physiology. Cadherin is a calcium-dependent cell-cell adhesion glycoprotein crucial for alveolar epithelial cells differentiation in lactating mammary gland as well as involution of mammary gland after weaning (Boussadia et al., 2002).

Overall, the GO terms underlying cell proliferation and immune systems were enriched in Kangayam cattle, and the same was also supported by QTL and gene annotation in underlying ROH regions contributing to health and carcass traits. Kangayam cattle, being active, powerful and highly prized draft animals, had a good capacity for agricultural operations and transport. Higher cell proliferation and stronger immune system are considered to be the prerequisite of better draft ability to combat stressful conditions as well as wear and tear. Due to continuous selection for the draft ability traits over generations, these animals might have gathered putative signatures in the genomic regions responsible for these traits. There were significant ROH differences at chromosome 3 and 5 between large and short statured breeds (**Table 4**). The genes identified in these regions were *PTGFR* and *HMGA2* responsible for the milk production and stature, respectively.

### Structuring of Cattle Breeds

The PCA based on the consensus ROH regions resolved the differences between breeds. The draft and short statured breeds were quite distinct from other breeds. Dairy and dual breeds also had their own identities and could not be clustered together. It was also interesting to note that the structuring of these cattle does remain unaffected when the number of consensus regions were scaled down to just 10 from 170 based on their contribution to the total variance. However, based on entire SNP dataset, Vechur, Kangayam and Ongole clustered separately from rest of the breeds but all dairy breeds along with Hariana cattle clustered together (**Supplementary Figure S4**). Hence, ROH analysis revealed the functionality (dairy, dual, and draft) of zebu cattle in a better way compared to SNP dataset.

## Conclusion

In conclusion, our study highlights characterization of autozygosity in seven diverse Indian cattle breeds (*B. indicus*) where genome coverage is found to be almost equal in short (ROH >_1 Mb_) and long (ROH >_8 Mb_) ROH regions. The level of genomic inbreeding (*F_ROH_*) revealed that the breeds are mostly random bred and hence preserve sufficient genetic variability. The ROH regions observed in these cattle breeds were able to differentiate dairy and draft breeds as well as small stature cattle revealing selection/adaptive footprints. The selection signatures in and around genes responsible for milk production, immunity, stress tolerance, and small stature were identified in dairy, draft, and miniature cattle.

## Data Availability Statement

We have uploaded the data on ICAR-Krishi portal and is in public domain with the URL http://krishi.icar.gov.in/jspui/handle/123456789/31167.

## Ethics Statement

The animal study was reviewed and approved by ICAR-NBAGR IAEC.

## Author Contributions

SD, SS and IG conceived and supervised the study. AS, SS, IG, SD, AK, NK, AD, and SJ participated in the data analysis. IG, SS, and SD drafted the manuscript. All the authors have read and approved the final manuscript.

## Funding

This work was financially supported by the Department of Biotechnology (DBT), Govt. of India.

## Conflict of Interest

The authors declare that the research was conducted in the absence of any commercial or economic associations that could be construed as a potential conflict of interest.
