# Supplementary material for: Genome-Wide Runs of Homozygosity Revealed Selection Signatures in Bos indicus
Source: Front Genet. 2020 Feb 21;11:92. doi: 10.3389/fgene.2020.00092 (PMC7046685; doi:10.3389/fgene.2020.00092)
Supplement: Supplementary file 1 [file Presentation_1.pdf]

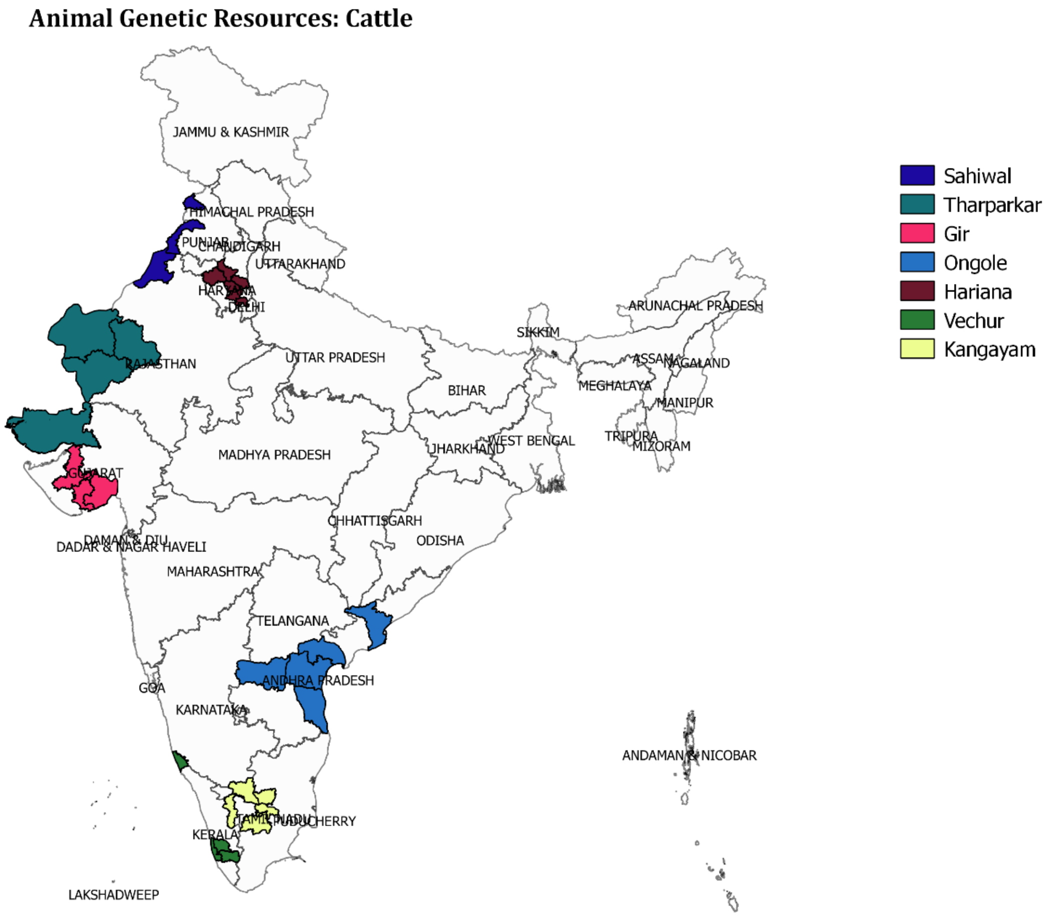

Supplementary Figure S1 | Location map of seven cattle breeds included in the study. 'Reproduced by permission of Surveyor General of India on behalf of Govt. of India under License No. BP15CDLA452. All rights reserved.'

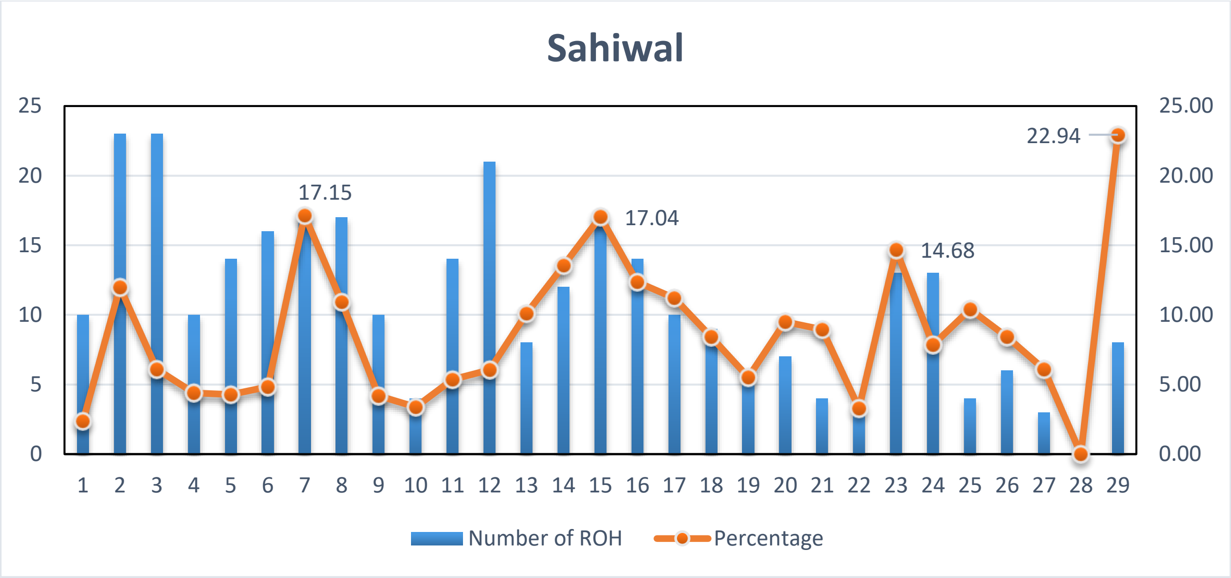
Sahiwal
17.15
17.04
14.68
22.94
Number of ROH
Percentage

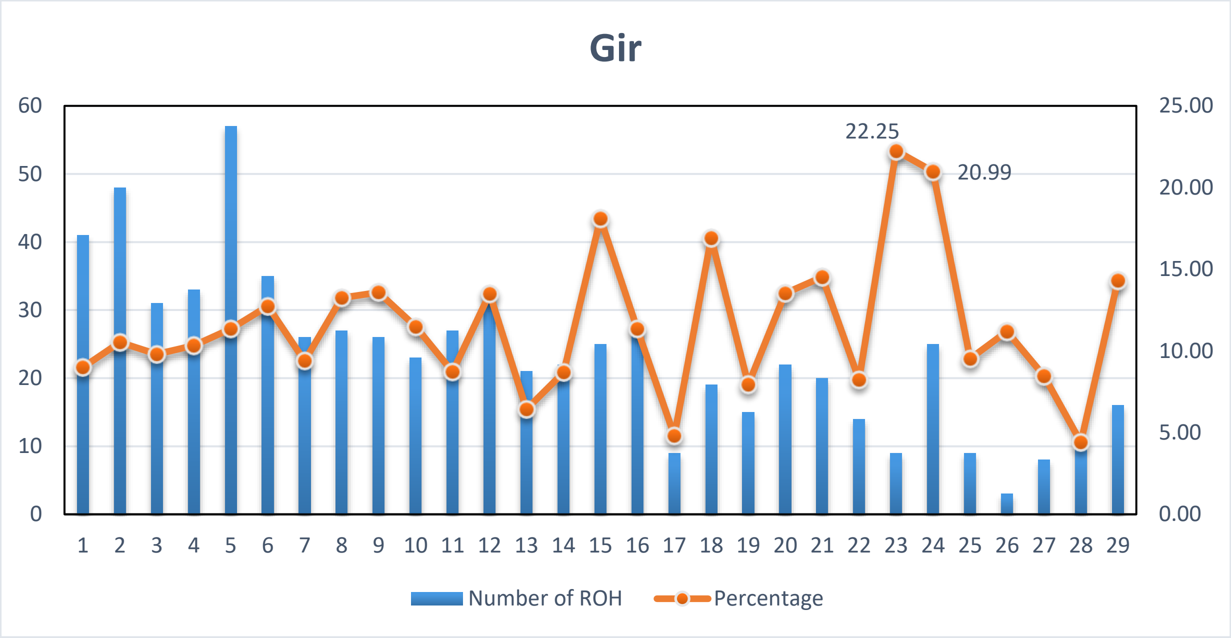
Gir
22.25
20.99
Number of ROH
Percentage

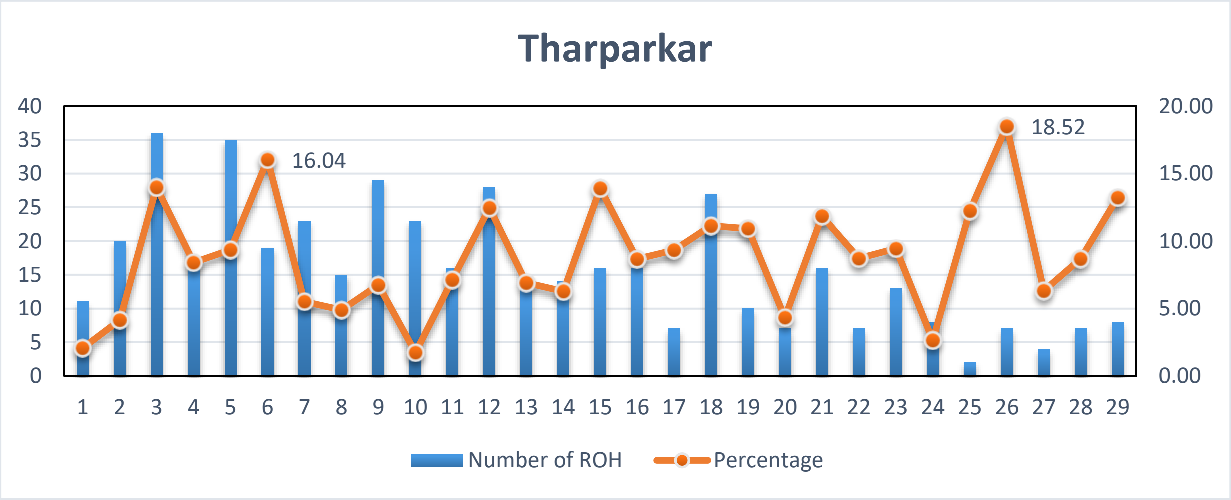
Tharparkar
16.04
18.52
Number of ROH
Percentage

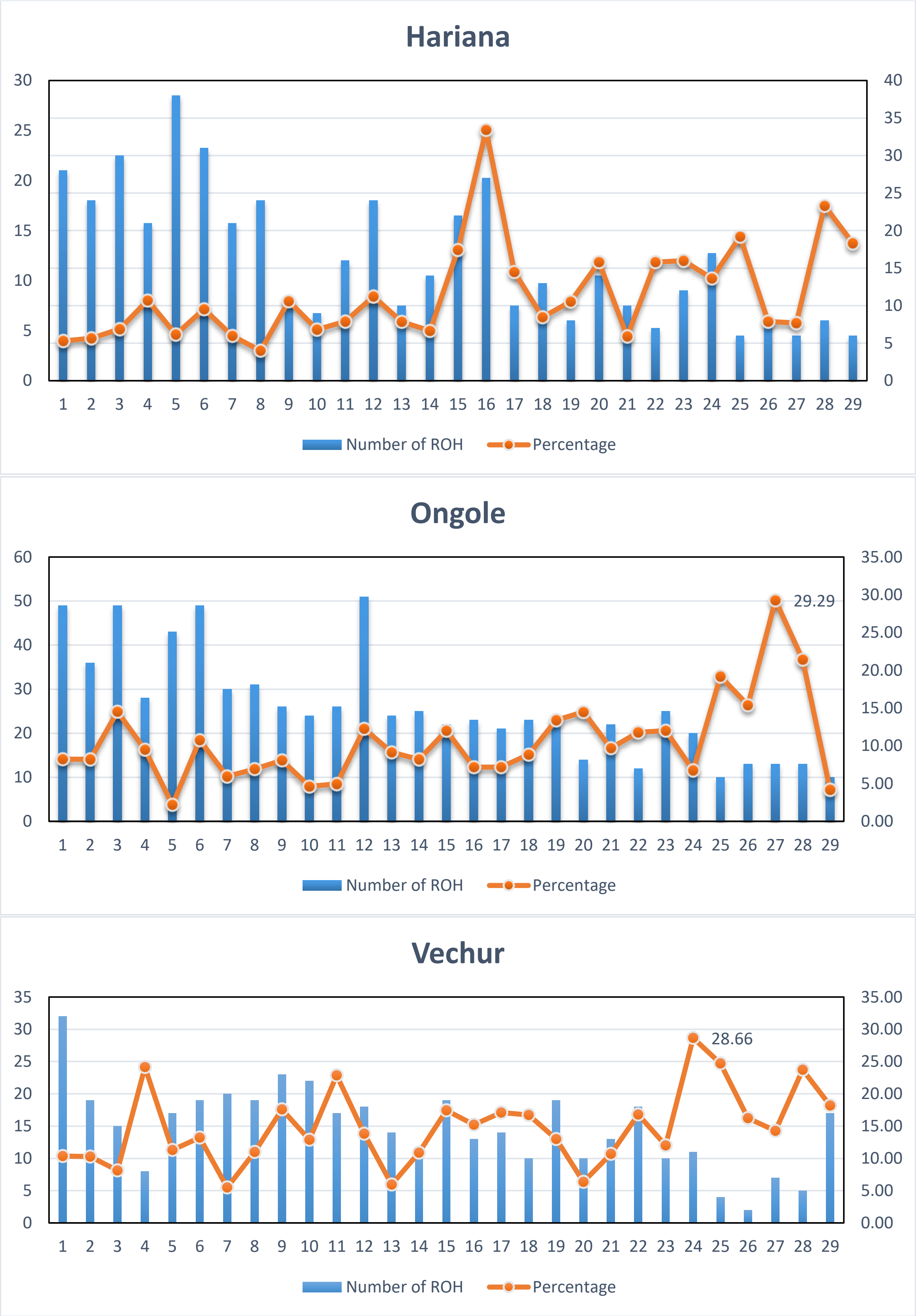

Supplementary Figure S2 | The number and percentage coverage of chromosomal length by ROH in different breeds.

(A)

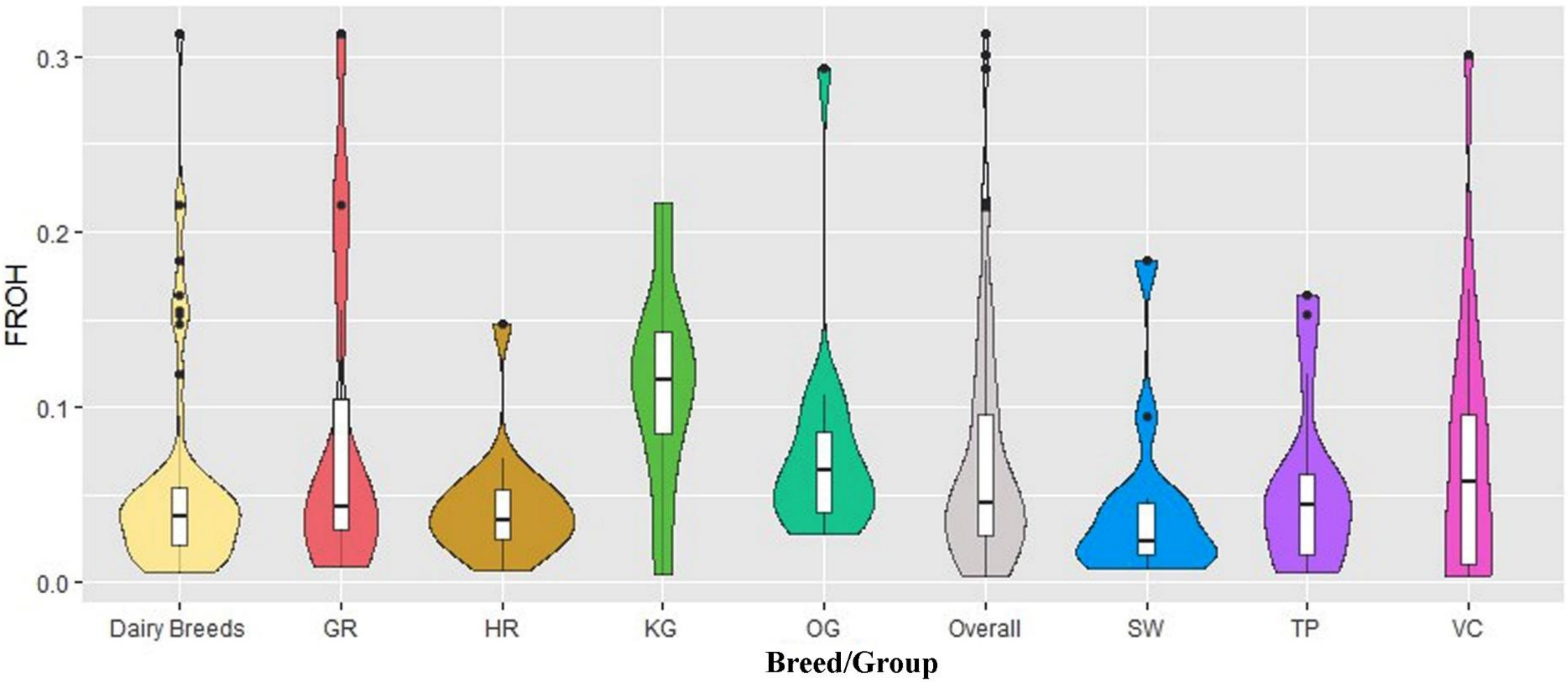
FROH
0.0
0.1
0.2
0.3
Dairy Breeds
GR
HR
KG
OG
Overall
SW
TP
VC
Breed/Group

(B)

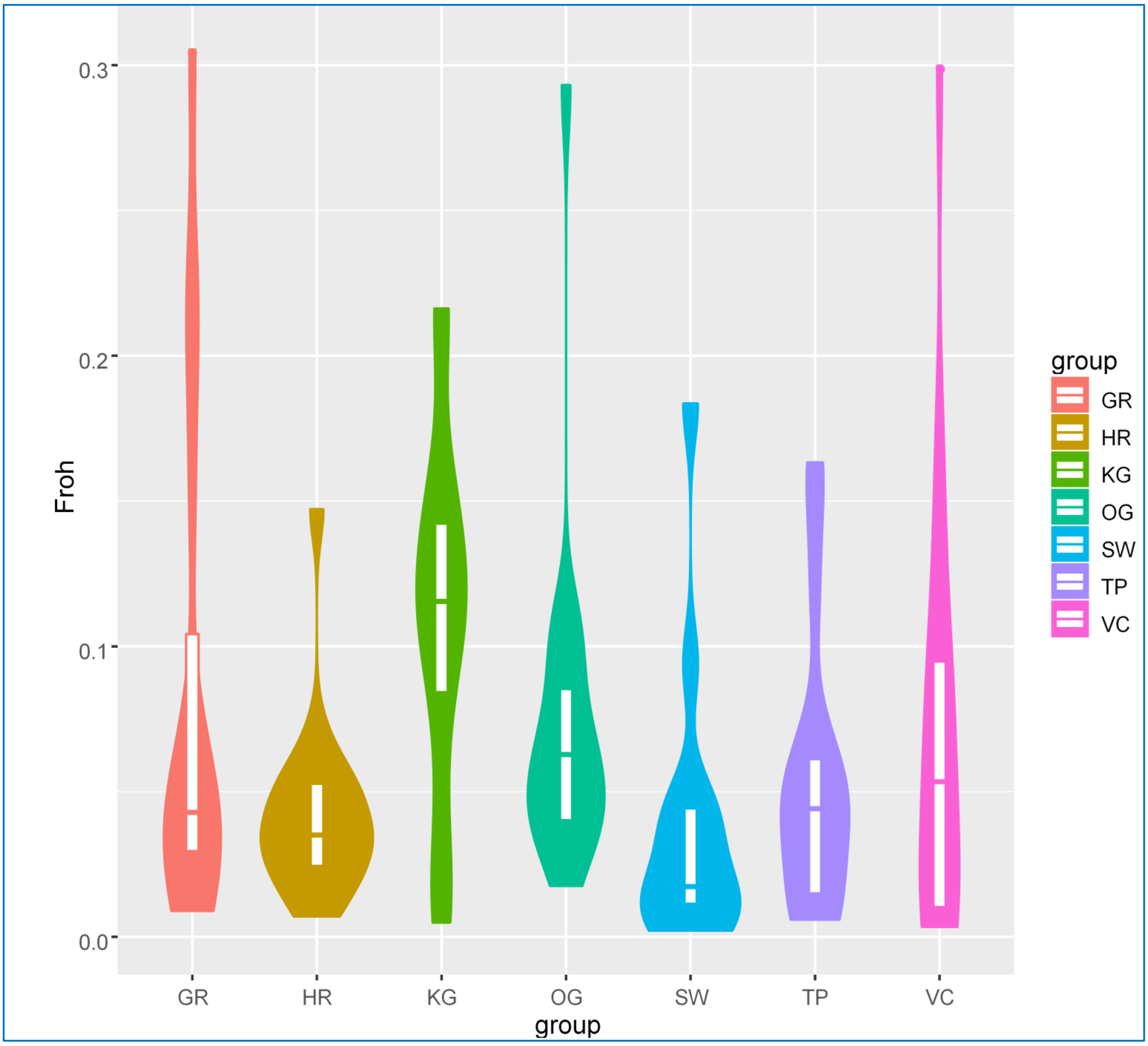

0.3
0.2
0.1
0.0
Froh
GR
HR
KG
OG
SW
TP
VC
group
group
GR
HR
KG
OG
SW
TP
VC

(C)

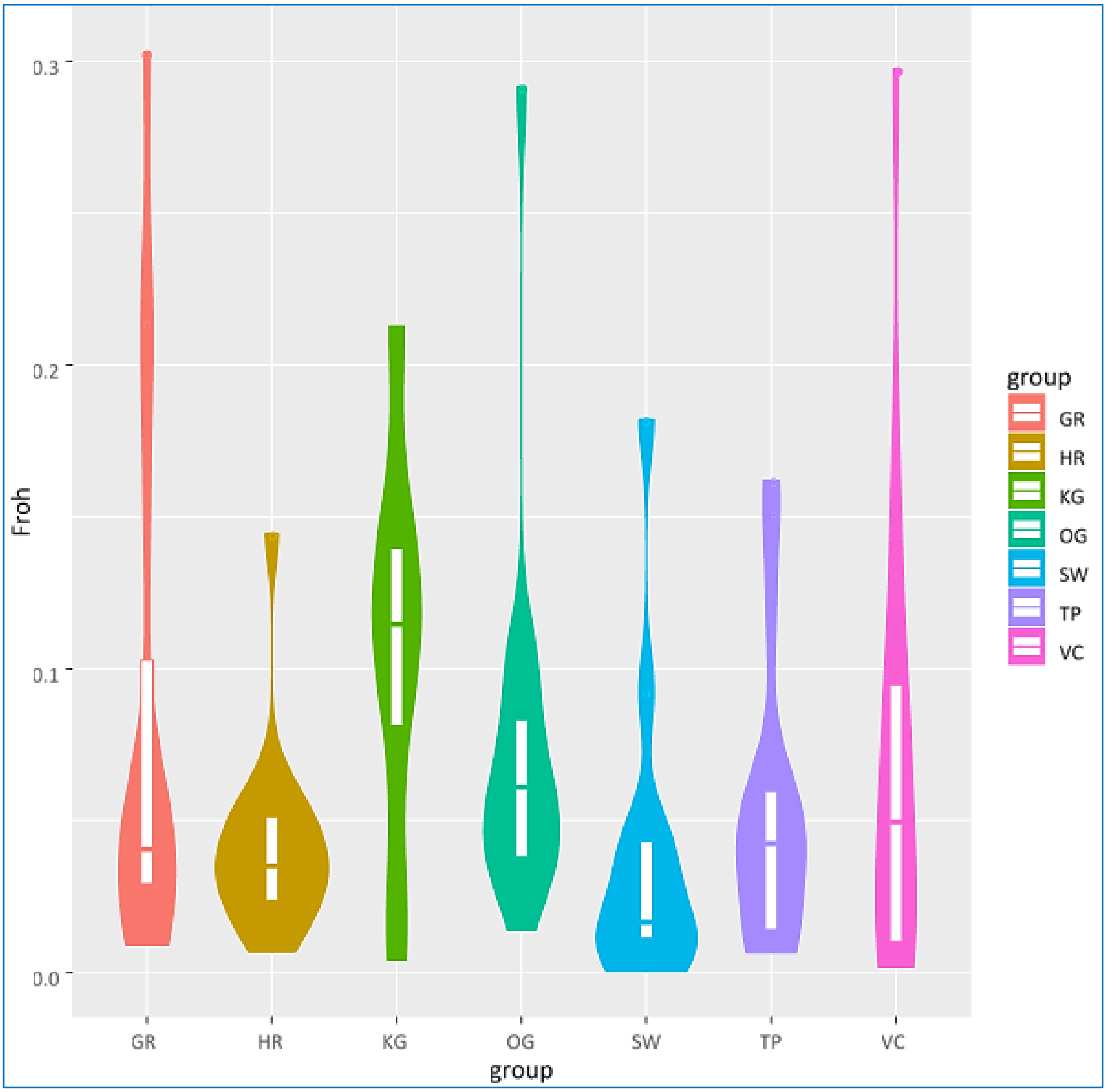

Supplementary Figure S3 | Plotting of genomic inbreeding (F_{ROH}) coefficient of seven Indian cattle breeds. Median value is depicted by horizontal line within box plot. (A) Plotting of breed-wise F_{ROH} obtained from software PLINK 1.9; (B) Plotting of breed-wise F_{ROH} values obtained from slidingRUNS option of R package "detectRUNS"; (C) Plotting of breed-wise F_{ROH} values obtained from consecutiveRUNS option of R package "detectRUNS".

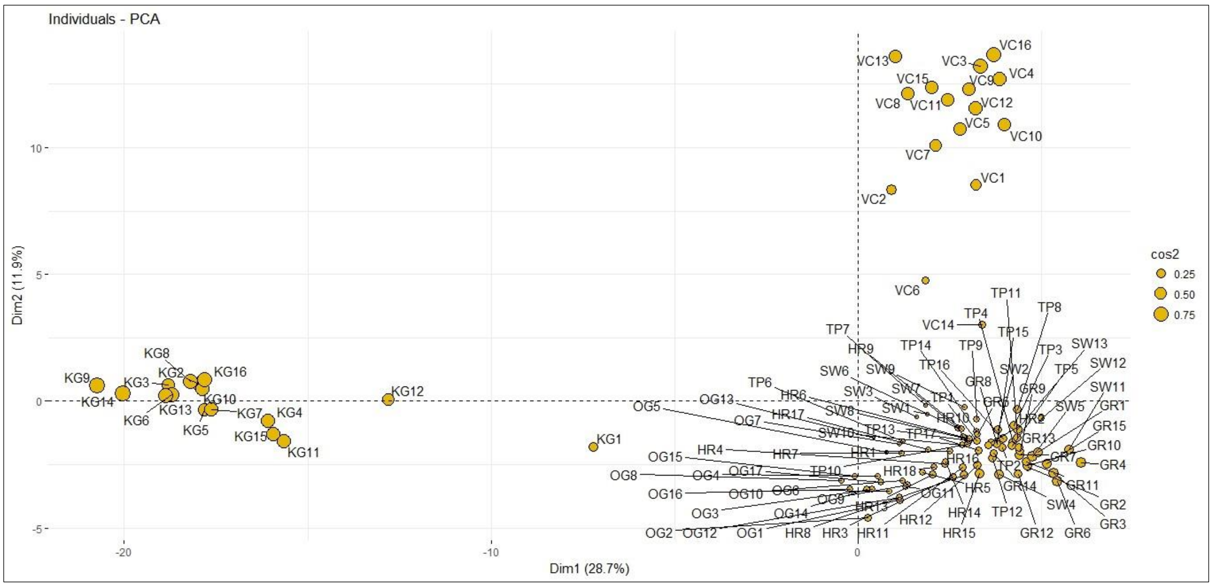

Supplementary Figure S4 | Principal Component Analysis based clustering of cattle breeds using entire SNP data.

(A) Dairy Group

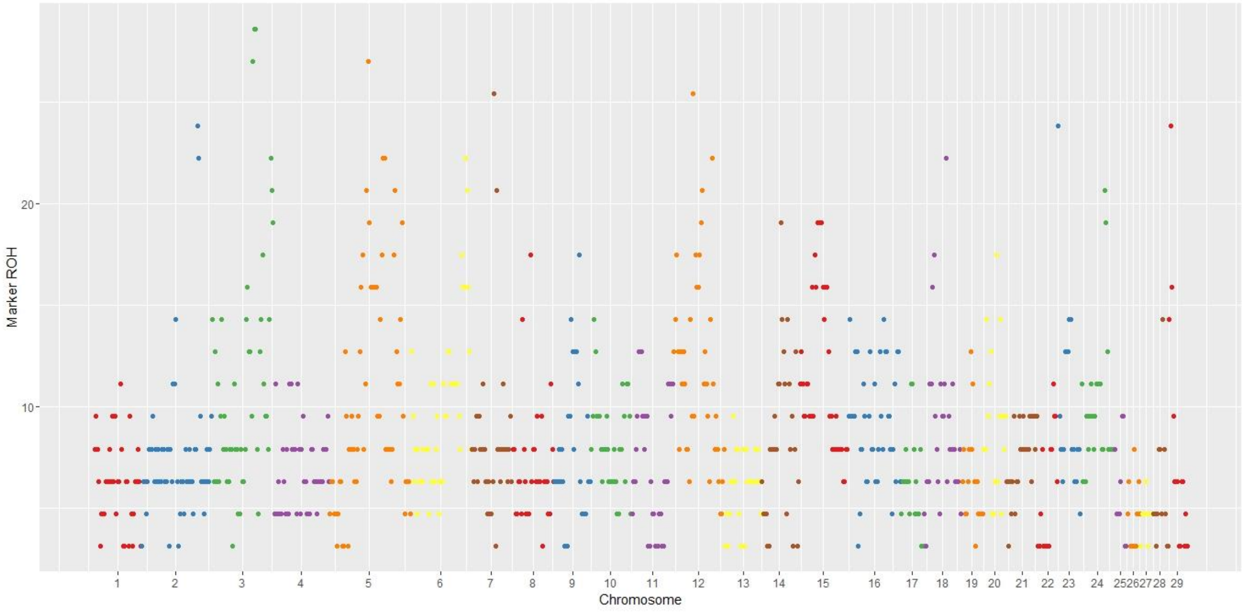

(B) Kangayam

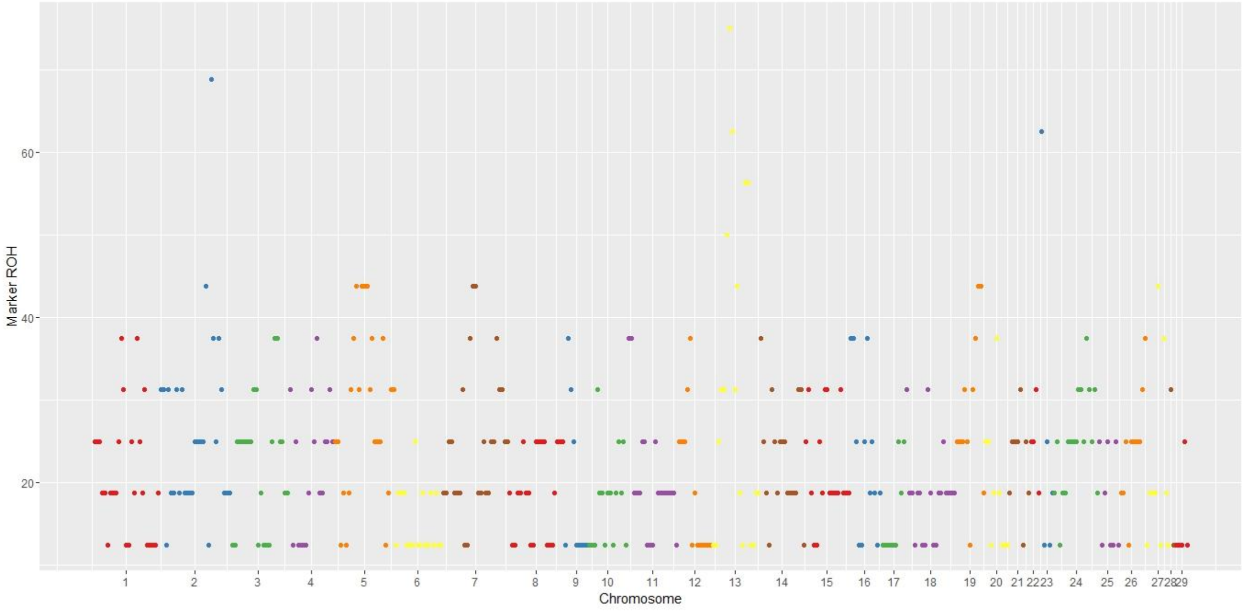

(C) Gir

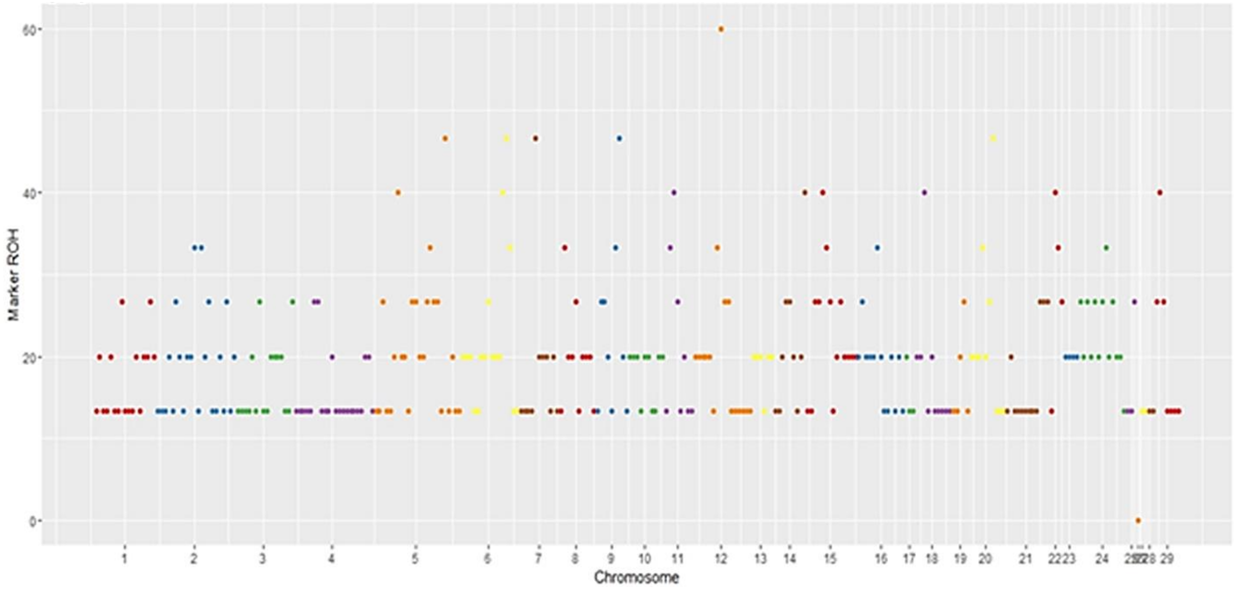

(D) Hariana

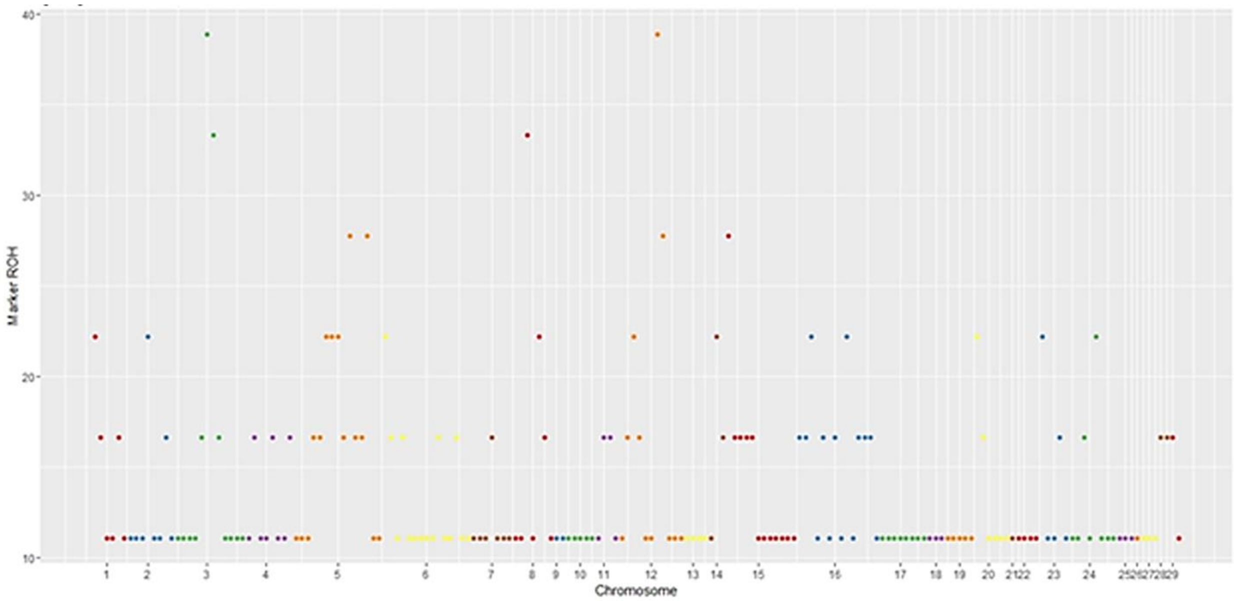

(E) Ongole

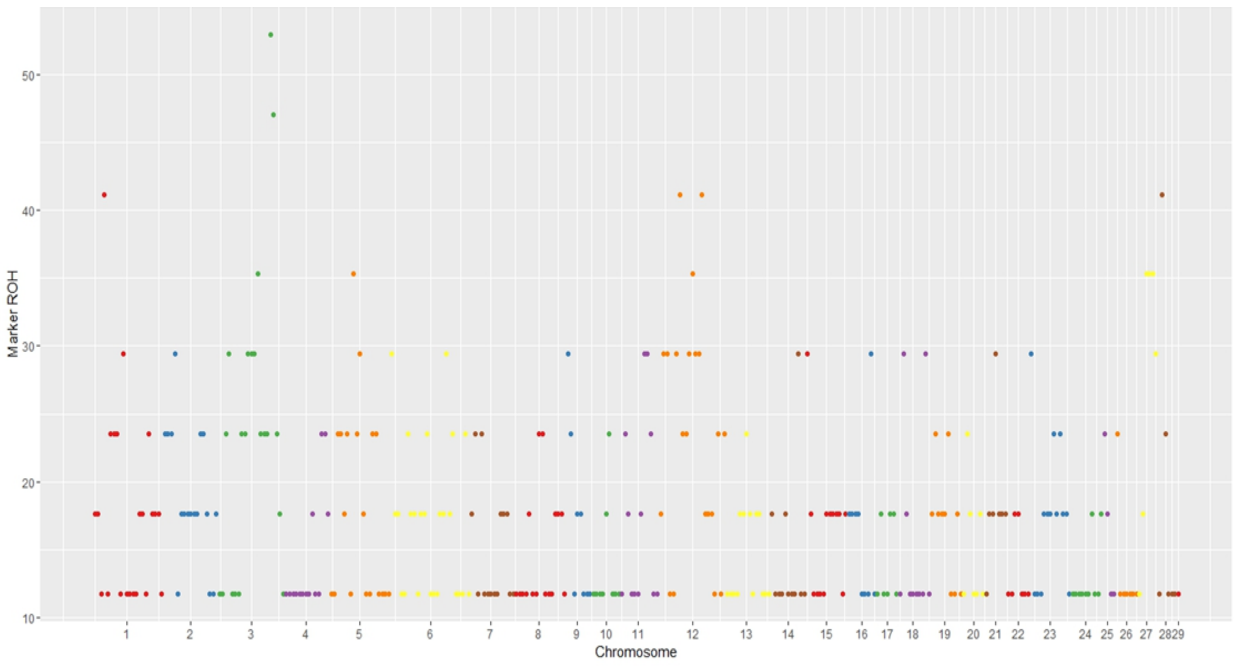

(F) Sahiwal

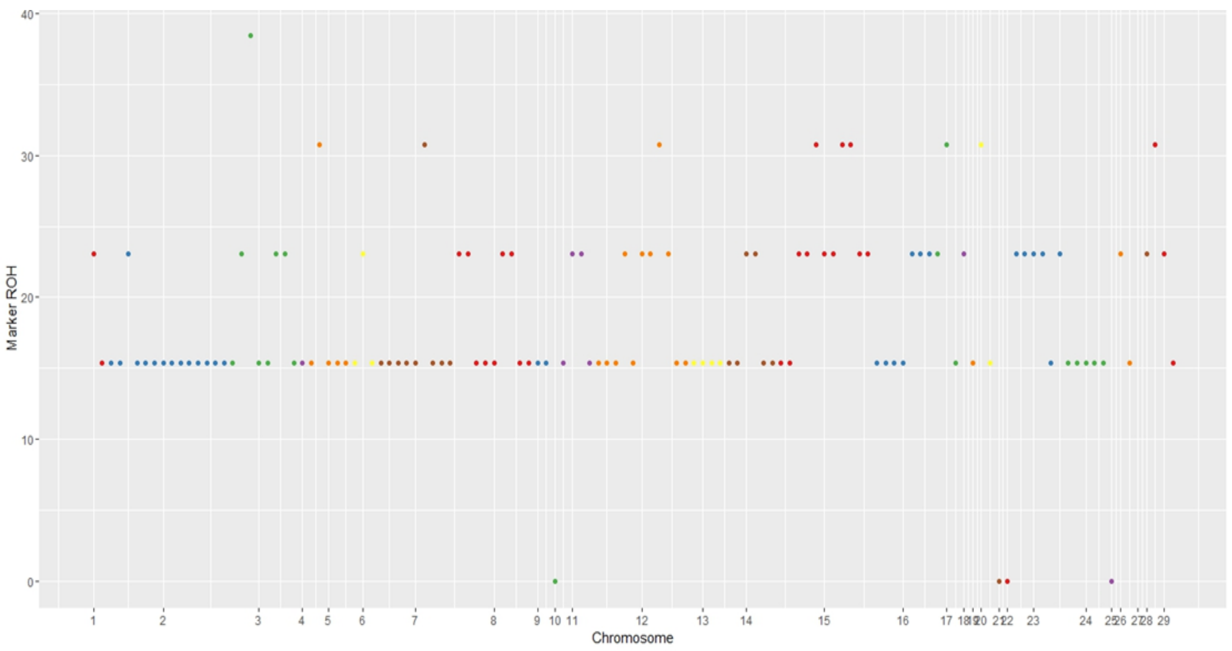

(G) Tharparkar

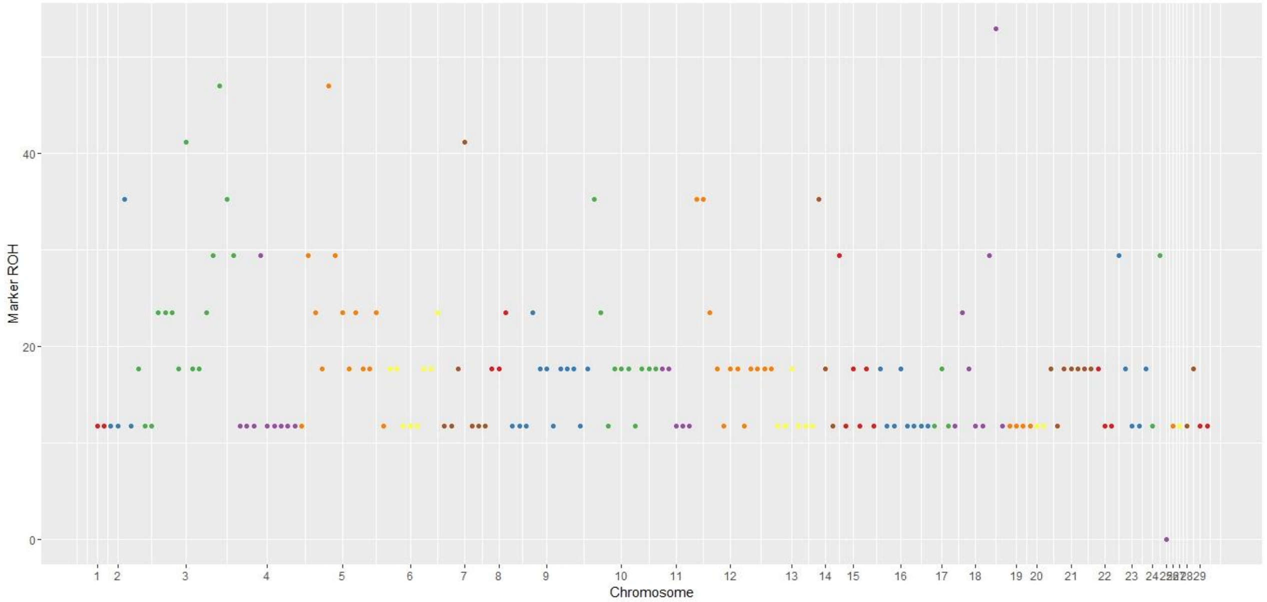

(H) Vechur

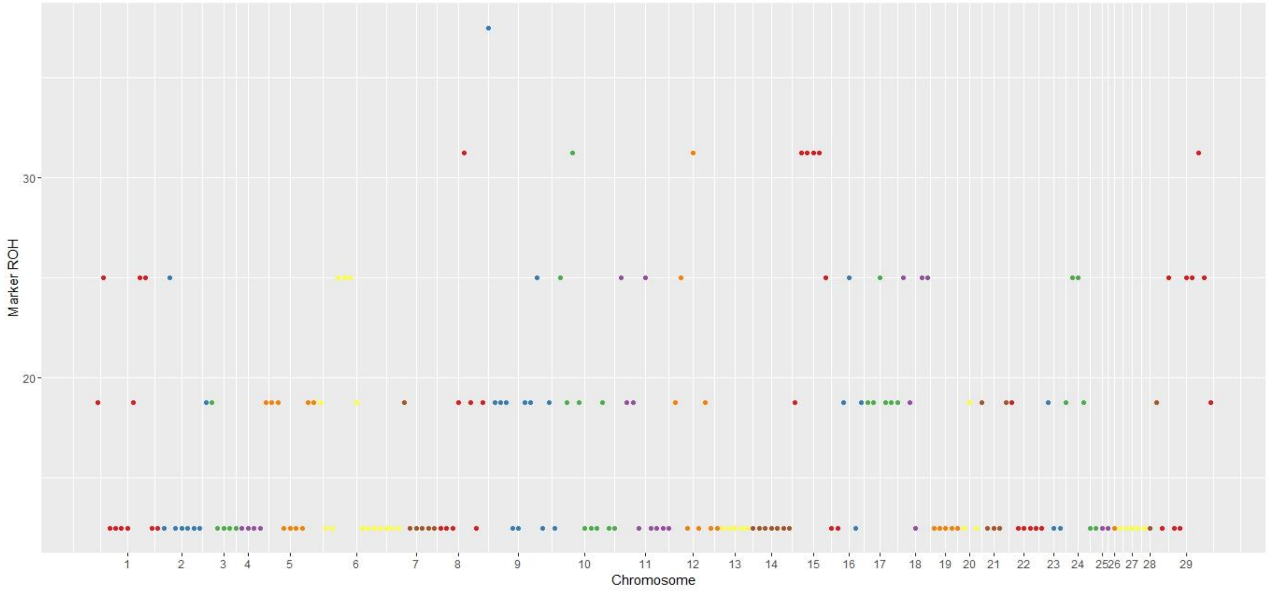

(I) Overall

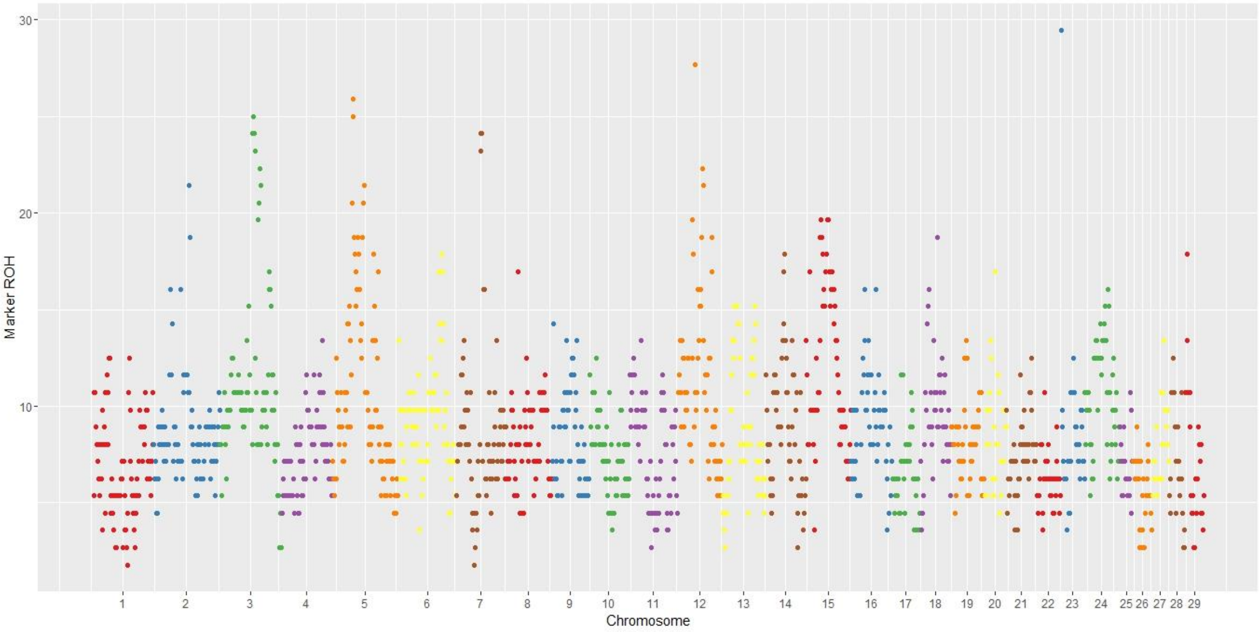

Supplementary Figure S5 | The Manhattan plots of overlapping ROH percentage in different breeds, dairy group and overall (A-I).

A

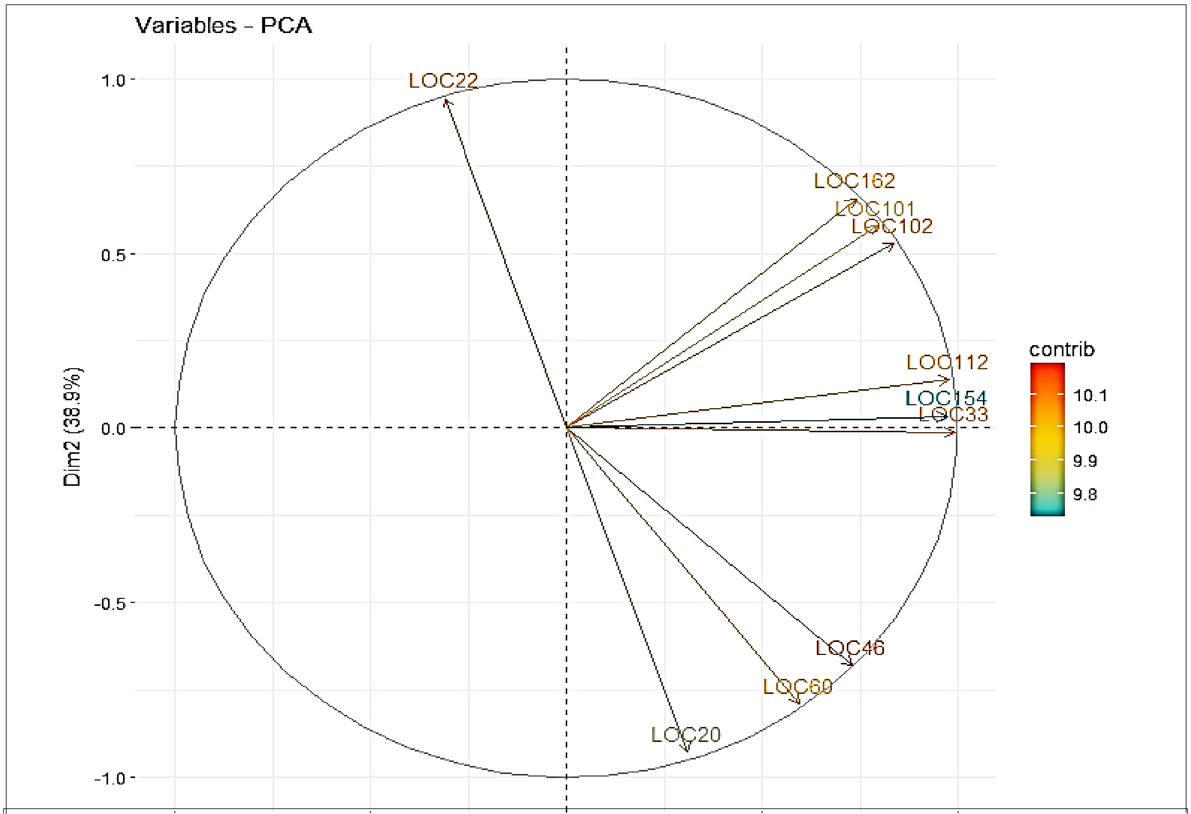

Rank	Locus (ROH region)	Chro	SNP1	SNP2	BP1	BP2
1	LOC112	5	BovineHD0500012478	BovineHD0500012478	43612254	43677680
2	LOC46	5	BovineHD0500014118	BovineHD0500014133	49025474	49082160
3	LOC33	5	BovineHD0500016309	BovineHD0500016331	57754977	57889506
4	LOC 154	5	BovineHD0500012579	BovineHD0500012843	43892509	44718715
5	LOC 20	5	BovineHD0500013550	BovineHD0500013566	47052115	47081107
6	LOC60	5	ARS-BFGL-NGS-34352	BovineHD0500014462	49842685	50295303
7	LOC 162	24	BTA-57747-no-rs	BovineHD2400007815	28582591	28817853
8	LOC 102	18	BovineHD1800004526	BovineHD1800004527	13724571	13730814
9	LOC 101	24	BovineHD2400008674	BovineHD2400008774	31889211	32178358
10	LOC22	15	BovineHD1500014141	ARS-BFGL-BAC-21165	48959770	49169653

B

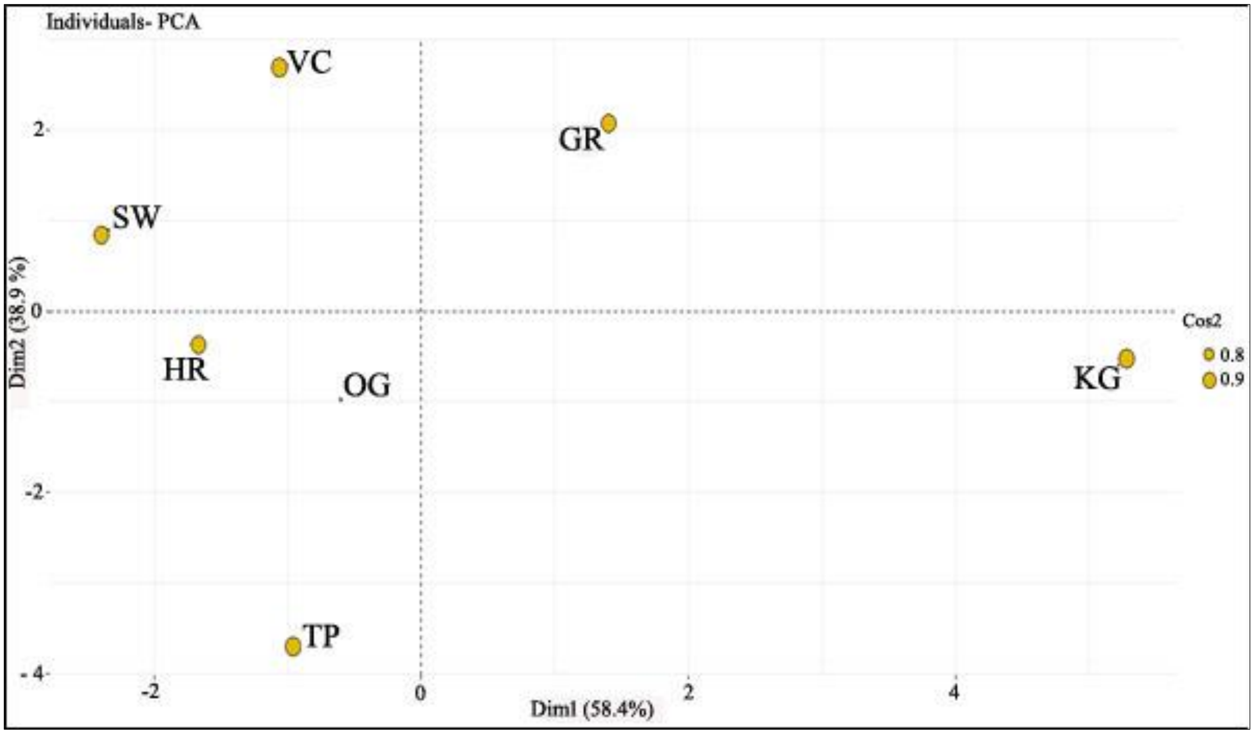

C

Principal component	Eigen value	Percentage contribution	Percentage Cumulative contribution
Dim.1	5.836462449	58.36462449	58.36462
Dim.2	3.894048405	38.94048405	97.30511
Dim.3	0.141904171	1.41904171	98.72415
Dim.4	0.091000973	0.91000973	99.63416
Dim.5	0.031984941	0.31984941	99.95401
Dim.6	0.004599061	0.04599061	99.95401

D

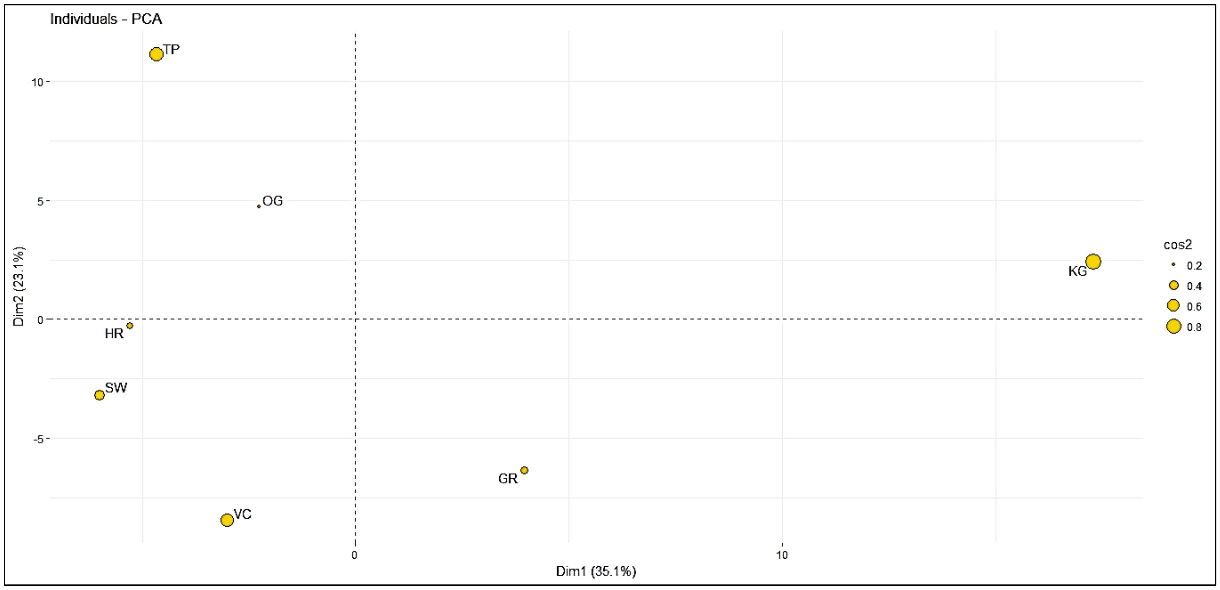

E

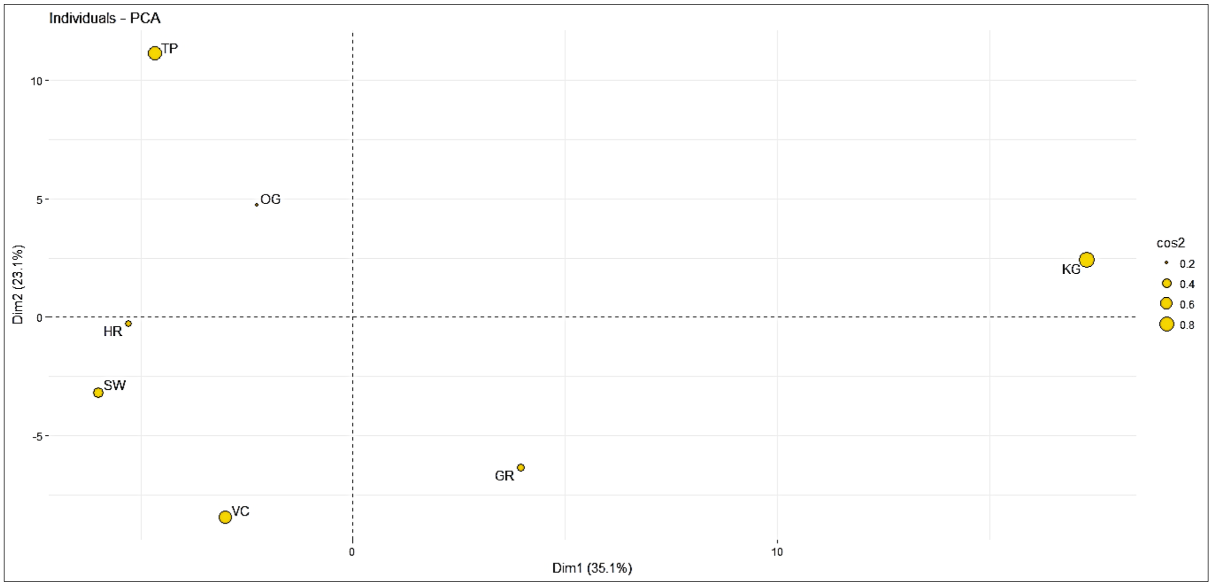

Supplementary Figure S6 | Principal Component Analysis based on frequencies of ROH regions of all breeds. (A) Contribution of top 10 consensus ROH regions; (B) Contribution of breeds based on top 10 loci; (C) Contribution of first six PCs based on top 10 loci, (D) Contribution of breeds based on top 92 loci; (E) Contribution of breeds based on top 170 loci. Point size represents contribution of the breed to the total variation (B-E). GR: Gir, HR: Hariana, KG: Kangayam, OG: Ongole, SW: Sahiwal, TP: Tharparkar and VC: Vechur.
