# Supplementary material for: Genome-Wide Runs of Homozygosity Revealed Selection Signatures in Bos indicus
Source: Front Genet. 2020 Feb 21;11:92. doi: 10.3389/fgene.2020.00092 (PMC7046685; doi:10.3389/fgene.2020.00092)
Supplement: Supplementary file 2 [file Table_1.docx]

**Supplementary Table S1|** Longest ROH segments and animals harbouring highest total ROH length in each of the seven Indian native cattle breeds (*Bos indicus*).

| Breeds | Longest segments | Animals with highest total ROH (Mb) |
| --- | --- | --- |
| Gir | 50.59 MB (7764 SNPs on Ch.12; GR 15)  46.12 Mb (8542 SNPs on Ch. 14; Gr 15)  43.79 MB (8682 SNPs onn Ch.20 ; GR 36) | GR15: 786.86  Gr36: 541.31  Gr38: 541.07 |
| Haryana | 48.29 MB (9341 SNPs on Ch. 17; HR 12)  45.47 MB (8221 SNPs on Ch. 12; HR 7)  36.18 MB (7412 SNPs on Ch.20; Hr 12) | HR12: 369.65  HR2: 175.83  HR7: 139.7 |
| Kangayam | 49.54 MB (8471 SNPs Ch.15; KG2)  41.90 MB (8900 SNPs, Ch.8; KG2)  36.97 MB (6717 SNPs, Ch.27; KG2) | KG16: 543.81  KG2: 537.19  KG20: 388.29 |
| Ongole | 68.17 MB (12610 SNPs, Ch.3; OG8)  37.01 MB (7943 SNPs, Ch. 8; OG8)  36.88 MB (6920 SNPs, Ch.27; OG14) | OG8:737.62  OG22: 267.55  OG4: 258.76  OG14:258.71 |
| Sahiwal | 47.76 MB (10392 SNPs, Ch. 7; SW2388)  45.79 MB (9006 SNPs, Ch.14; SW2388)  35.72 MB (6164 SNPs, Ch.15; SW2388) | SW2388: 461.13  SW2004:236.41  SW242:119.11 |
| Tharparkar | 80.22 MB (17050 SNPs, Ch.6; TP7536)  47.76 MB (7759 SNPs, Ch.12; TP7487)  41.39 MB (8980 SNPs, Ch. 6; TP6960) | TP7536: 410.1  TP787: 383.4  TP6960: 299.54 |
| Vechur | 61.26 MB (12018 SNPs, Ch.14; VC1)  50.69 MB (9579 SNPs, Ch.18; VC18)  50.58 MB (7763 SNPs, CH. 4; VC1) | VC1: 756.94  VC18: 420.07  VC7: 238.72 |
