# Supplementary material for: Genome-Wide Runs of Homozygosity Revealed Selection Signatures in Bos indicus
Source: Front Genet. 2020 Feb 21;11:92. doi: 10.3389/fgene.2020.00092 (PMC7046685; doi:10.3389/fgene.2020.00092)
Supplement: Supplementary file 4 [file Table_3.docx]

**Supplementary Table S3 |** Detailed functions of genes identified in top 20 ROH islands (±2MB) in dairy and draft breeds.

| **Type of breed** | **Gene identified** | **Important function** |
| --- | --- | --- |
| **Dairy breed** | PTGFR (Prostaglandin F Receptor) | Contributes to the regression of the corpus luteum and thereby the estrus cycle in many species of farm animals and induce labor in pregnant farm animals |
|  | ZAR1L (Zygote arrest-1 like) | Predominantly expressed in oocytes and early embryos. Its protein functions as an RNA regulator in early embryos. |
|  | IFI44 (interferon-induced protein 44) | Combats the hepatitis C and D virus infection. |
|  | HELB (DNA helicase B) | Encodes a DNA-dependent ATPase responsible for unwinding of DNA necessary for DNA replication, repair, recombination, and transcription. |
|  | CSN1S1 (Casein Alpha S1), CSN2 (casein beta), CSN1S2 (Casein Alpha S2), and CSN3 ([casein kappa](https://www.genenames.org/data/gene-symbol-report/#!/hgnc_id/HGNC:2446)) | Important genes related to milk production/ constituent |
|  | GNRHR (Gonadotropin Releasing Hormone Receptor) | Important candidate gene for the reproductive traits. |
| **Draft breed** | GPX4 (Glutathione Peroxidase 4) | Catalyzes the reduction of peroxides and thereby protects cells against oxidative damage |
|  | SVIL (Supervillin) | 205-kD F-actin–binding protein in bovine neutrophils, has an important role in body defense mechanism. |
|  | LYZL1 (Lysozyme like1) | Lysozyme like activity is responsible for the defense response to gram positive and gram negative bacteria. |
|  | COMMD1(Copper metabolism domain containing 1) | Copper ion binding protein |
|  | CCT4 (Chaperonin Containing TCP1 Subunit 4) | Encodes chaperonin containing TCP1, subunit 4 (delta) involved in protein folding |
|  | ALGS (Alagille syndrome) | Involved in the intercellular signaling pathway to regulate interactions between physically adjacent cells |
|  | SMAD9 (SMAD family member 9) | Involved in transforming growth factor beta receptor signaling pathway |
|  | FGF22 (Fibroblast Growth Factor 22) | Plays an important role in cell growth, hair follicle development, morphogenesis and tissue repair. |
|  | HSF2BP (heat shock transcription factor 2 binding protein), HSPA14 (heat shock protein family A (Hsp70) member 14) & HSPA4 (heat shock protein family A (Hsp70) member 4) | Involved in heat stress response. |
|  | IL23A (Interleukin 23 Subunit Alpha) | Important role in immune system (increases angiogenesis and osteoclast genesis) |
