# Supplementary material for: Genome-Wide Runs of Homozygosity Revealed Selection Signatures in Bos indicus
Source: Front Genet. 2020 Feb 21;11:92. doi: 10.3389/fgene.2020.00092 (PMC7046685; doi:10.3389/fgene.2020.00092)
Supplement: Supplementary file 5 [file Table_4.docx]

**Supplementary Table S4 |** Details of the genes in the GO enriched terms and pathway analysis in dairy and draft breeds.

| **Gene group enriched** | **Gene** | **Important functions** |
| --- | --- | --- |
| **Dairy breed** | | |
| Mammary gland development | Kappa-casein (*CSN3*) and/ *COP9* signalosome complex subunit 3 (*COPS3*) | The protein encoded by this gene possesses kinase activity that phosphorylates regulators involved in signal transduction. |
| Steroid metabolic process | *RDH5* (11-cis retinol dehydrogenase), *SDR9C7* (Short-chain dehydrogenase/reductase family 9C member 7), *HSD17B6* (17-beta-hydroxysteroid dehydrogenase type 6), *RDH16* (Microsomal NAD+dependent retinol dehydrogenase 4), *CYB5B* (Cytochrome B5 TYPE B), *HSD11B2* (Corticosteroid 11-beta-dehydrogenase isozyme 2), *GPX7* (Glutathione peroxidase 7), *GPX4* (Glutathione peroxidase 4), *LCAT* (Lecithin-cholesterol acyltransferase) and *PLA2G15* (Group XV phospholipase A2) | These proteins mainly have dehydrogenase, peroxidase, acetyltransferase and oxidase activities. |
| Cell junctions | Cadherin (CADH1 CADH3, CADH5 and CADH11)  Myosin (MYO1A and MYO1B) | Cadherin is a calcium-dependent cell-cell adhesion [glycoprotein](https://en.wikipedia.org/wiki/Glycoprotein). E-cadherin is essential for the differentiation of alveolar epithelial cells in the lactating mammary gland. Myosin is actin binding motor protein that interacts with F actin filaments. |
| Golgi lumen | *AGRP* (Agouti-related protein), *CSN2* (Beta-casein), *CSN1S2* (Alpha-S2-casein), *CSN3* (Kappa-casein) and *AGRP* (Agouti-related protein). | *AGRP* is a signaling molecule, *CSN2*, *CSN1S2* and *CSN3* are milk proteins which have significant influence on milk production and constituent traits. |
| **Draft breed** | | |
| Microtubules | *TUBB1, KIF3B, TPX2, AURKA, TTLL9, MAPRE1, APC2, DCTN6 and KIF5A* | Involved in cytoskeleton structuring and microtubular functions. |
| Catalytic activity acting on RNA | LYZL1 (Lysozyme like1), *CYB561* (CytochromeB561) and *GSR* (glutathione reductase, mitochondrial) | LYZL1 (Lysozyme like 1) has an important role in immunity, body defense mechanism and disease resistance. *CYB561* (CytochromeB561) and *GSR* (glutathione reductase, mitochondrial), have oxidoreductase activity and antioxidant property, respectively.  Antioxidants could help to mitigate any form of oxidative/nitrosative stress or its consequences. |
| Activation of pre-replicative complex | *PRIM1*/2 (DNA Primase), *MCM6*/*8*/*10* (DNA helicase) and *CDK2* (Cyclin-dependent kinase 2) and *ORC4* (Origin recognition complex subunit 4). | These genes are involved in DNA replication and cell proliferation. |
| G2/M transition | *PSMC5* (26S protease regulatory subunit 8), *AURKA* (Aurora kinase A), *PSMF1*(Proteasome inhibitor PI31 subunit), *NEDD1*(Protein NEDD1), *CEP131*(Uncharacterized protein; Centrosomal Protein of 131 family), *PPP2CB* (Serine/threonine-protein phosphatase 2A catalytic subunit beta isoform), *DCTN2* (Dynactin subunit 2), *SKP1* (S-phase kinase-associated protein 1), *MAPRE1* (Microtubule-associated protein RP/EB family member 1), *PSMD12* (26S proteasome non-ATPase regulatory subunit 12), *CSNK1D* (Casein kinase I isoform delta), *PPP2CA* (Serine/threonine-protein phosphatase 2A catalytic subunit alpha isoform), *OPTN* (Optineurin), *CEP250* (Uncharacterized protein; centrosomal Protein of 250 family), *TPX2* (Targeting protein for Xklp2), *CDK2* (Cyclin-dependent kinase 2). | Involved in the cellular process, cell cycle, catabolic process, chromatin organization, chromosome segregation, catabolic process, apoptotic process and immune system process (*PPP2CA*, I-Kappa beta Kinase, NF-K-beta cascade (*OPTN*). |
